# Direct reprogramming of epidermal cells toward sweat gland-like cells by defined factors

**DOI:** 10.1038/s41419-019-1503-7

**Published:** 2019-03-20

**Authors:** Bin Yao, Jiangfan Xie, Nanbo Liu, Tian Hu, Wei Song, Sha Huang, Xiaobing Fu

**Affiliations:** 1 Wound Healing and Cell Biology Laboratory, Institute of Basic Medical Sciences, General Hospital of PLA, Beijing, 100853 P.R. China; 2 Key Laboratory of Tissue Repair and Regeneration of PLA, and Beijing Key Research Laboratory of Skin Injury, Repair and Regeneration, Fourth Medical Center of PLA General Hospital, Beijing, 100048 P.R. China; 3 Burn Department of the First People’s Hospital of Zhengzhou City, Zhengzhou, 450004 P.R. China; 4 Department of Cardiac Surgery, Affiliated South China Hospital, Southern Medical University (Guangdong Province People’s Hospital), Guangzhou, 510515 P.R. China; 5 School of Medicine, Nankai University, Tianjin, 300052 P.R. China

## Abstract

Several studies have reported inducing adult cells into sweat gland-like cells; however, slow transition and low efficiency limit the potential for cell-based treatment. Here, we show that overexpression of the transcription factor FoxC1 was sufficient to reprogram epidermal cells to induced functional sweat gland-like cells (iSGCs). The iSGCs expressing secreting-related genes, had a global gene expression profile between fetal SGCs (P5) and adult SGCs (P28). Moreover, iSGCs transplanted into the burn mice model facilitated wound repair and sweat gland regeneration. We further demonstrated that the Foxc1 upregulated BMP5 transcription and BMP5 is responsible for the cell-type transition. Collectively, this study shows that lineage reprogramming of epidermal cells into iSGCs provides an excellent cell source and a promising regenerative strategy for anhidrosis and hypohidrosis.

## Introduction

Millions of sweat glands (SGs) are spread over the human body and sweating is indispensable for the maintenance of human body temperature^1^. Anhidrotic/hypohidrotic ectodermal dysplasia patients, who lack sweat glands, would suffer the relative risk of heat stroke and potentially death on heat stress^2,3^. Sweat gland deficiency or sweating disability may be therapeutically achieved by stimulation of endogenous regeneration or transplantation of mesenchymal stem cells, whereas the endogenous progenitors appear to be limited in both their mitotic competence and differentiation, and the efficacy of cell transplantation therapies has been limited by poor transition rates of trans-lineage and long treatment duration^4,5^. Thus, we thought that sweat gland cells (SGCs) transplantation may be a potential solution without these limits.

Because the unavailability of normal skin tissues containing sweat glands and manual experimental skills of SGC isolation from skin tissue were complex, they are difficult to culture in large numbers in vitro^6,7^. Fortunately, the discovery of induced pluripotent stem cells (iPSCs) that allows the direct generation of specific cell types from differentiated somatic cells by overexpression of lineage-specific factors has recently emerged as a promising renewable source that can be used to generate SGCs^8^. Although various cell types have been used to induce SGC differentiation through SG cells coculture or SG conditional medium^9,10^, in vitro SG reprogramming was barely concerned. Someone converted fibroblasts into SGCs successfully with TF NF-kB and Lef-1, but the inductive efficiency is limited and the underlying mechanism is unrevealed^11^. As both epidermal cells (ECs) and SGCs were developed from epidermal progenitors, the similarity between ECs and SGCs on the molecular level implicated further that ECs are a potential cell source for SGC reprogramming and in vivo treatment of a large scale of traumatic injury recovered with less effort of lineage transition^12,13^.

According to previous studies, mouse genetic models find sweat gland development initiated by coordination of BMP4, BMP5, and FGF18^14^ and Wnt, Eda, and Shh signaling pathways are correlated with morphological stages in gland formation and sweating function^15^.

In this study, we sought to identify and characterize the transcription factors (TFs) that govern sweat gland specification. We compared the transcriptional profile of ECs and SGCs to screen instructive TFs and demonstrated that a specific combination of three transcription factors, FoxC1, Irf6, and Tcfp2l1, and a single transcription factor, FoxC1 was sufficient to generate functional SGCs directly from ECs that expressed SG-specific markers and SG-like gene signature. Furthermore, we found that induced SGC transplantation was capable of facilitating the sweat gland repair in vivo. Finally, we elucidated the molecular mechanism of FoxC1 specifying the SGC differentiation through activating the expression of BMP5 and promoted sweat gland fate.

## Materials and methods

### Mouse manipulations

Mice were all C57BL/6 genetic background. For the paw pad burn model, mice were anesthetized with Pentobarbital (100 mg/kg) and received preoperative subcutaneous Buprenorphine (0.1 mg/kg). Second-degree burn was administered to back paw pads. Mice recovered in clean cages with paper bedding to prevent irritation or infection. Mice were monitored daily and killed at specified times post wounding.

### Epidermal and sweat gland cells isolation

Epidermal and sweat gland cells were isolated as previously described^16^. Epidermal cells isolation mice were killed and the back skin was diced to pieces. Then 2 mg/ml Dispase was digested overnight at 4 °C, separated the epidermis and dermis, and discarded the dermis. Minced epidermis and 0.05% Trypsin-EDTA were digested for 20 min at 37 °C. The suspension passed the cell strainer (FALCON, 40-μm Nylon 352340), and was centrifuged at 1000 rpm for 5 min. All operations were perfomed under sterile conditions.

Sweat gland cells isolation footpads were cut down and diced to pieces. The pieces were digested in 2 mg/ml Collagenase I for 2 h and aspirated the sweat gland cells with a micropipette (Eppendorf, Germany). Sweat gland cells specific medium contains 50% DMEM (Gibco, New York, NY) and 50% F12 (Gibco) supplemented with 5% fetal calf serum (Gibco), 2 ng/mL liothyronine sodium (Gibco), 1 mL/100-mL penicillin–streptomycin solution, 1 mL/100-mL insulin–transferrin–selenium (Gibco), 0.4 µg/mL hydrocortisone succinate (Gibco), and 10 ng/mL epidermal growth factor (Peprotech, Rocky Hill, NJ). All operations were performed under sterile conditions.

### Histology and immunofluorescence

Tissue specimens were fixed in 4% PFA and embedded in paraffin. Antigen retrieval of samples was carried out in 10 mM citric acid buffer (pH 6.0 + 0.1) for 11 min using a microwaver (Midea). Blocking for 1 h with goat plasma and antibody Diluent (Beyotime) was followed by overnight incubation at 4 °C with the following primary antibodies: rabbit polyclonal anti-cytokeratin-5 (Abcam, 1:300), rabbit polyclonal anti-cytokeratin-14 (Abcam, 1:300), rabbit polyclonal anti-cytokeratin-8 (Abcam, 1:300), mouse polyclonal anti-cytokeratin-18 (Abcam, 1:300), rabbit polyclonal anti-S100 (Abcam, 1:300), mouse polyclonal anti-CGRP (Abcam, 1:300), and rabbit polyclonal anti-Na^+^–K^+^–ATPase (Abcam, 1:300). Detection was carried out using donkey anti-rabbit/mouse IgG (H+L) conjugated with Alexa Fluor 488 (both 1:300) for immunofluorescence (IF) microscopy. Cell immunofluorescence staining samples were fixed in 4% paraformaldehyde for 15 min, and then the rest of the steps are performed as described above.

### RNA-seq and TF selection

Six littermate infant mice were prepared for cell isolation to balance the sample difference. RNA-seq was performed using HiSeq 2500 (Illumina). Transcription factors that were enriched over twofold of sweat gland cells vs epidermal cells were selected in our own data. Putative transcription factors were filtered by selecting genes with among a ‘GO cellular component term’ “nucleus”, a ‘GO molecular function term’ “DNA binding”, and ‘GO pathway terms’ “gland development” “epidermis development.” Data were then *z*-scored and plotted in R using the heatmap function of the gplots package (Fig. S1).

### Western blot

Cells were washed with cold PBS and lysed in RIPA buffer. Protein concentration was tested with the BCA Assay Kit (Bio-Rad, Richmond). Samples were denatured at 95 °C for 5 min, separated using SDS-PAGE, and transferred to polyvinylidene difluoride (PVDF) membranes (GE Healthcare, Piscataway, NJ, USA). After blockage with 5% BSA for 1 h at room temperature, the membrane was probed with rabbit polyclonal anti-FoxC1 (Abcam, 1:1000) overnight at 4 °C, then incubated with HRP-conjugated secondary antibody for 1 h, was visualized using the chemiluminescence system (Millipore), and exposed to X-ray medical films (Kodak/Carestream Health). Actin was used as a loading control.

### Cell proliferation and transitional ratio curve

Cell proliferation was evaluated through the CCK8 assay. Ten microliters of CCK8 working buffer was added into 96-well plates, tested with absorbance at 450 nm, and was measured using a Tecan Infinite M200 Pro microplate reader. Transitional rate was calculated through differential digestion. First, trypsinized transfected cells were washed with PBS twice for 1 min, then digested for another 3 min, and collected the cells for counting. The ratio of transfected cells number vs control is the transitional rate.

### Ca^2+^ absorption test

Fourteen days after transfection, the medium was changed to SG cell culture medium with 2 mM CaCl_2_ for at least 6 h, and then cells were loaded with Fluo 3/AM (Invitrogen) at a final concentration of 5 μM for 30 min at room temperature. The cells were washed with calcium-free PBS three times and acetylcholine (Ach, Sigma) (10 mM) was added to the medium. The Fluo 3 fluorescent signal was recorded using laser scanning confocal microscopy. The SG cells were used as a positive control.

### Sweat test

The footpads of anesthetized mice were first painted with 2% (w/v) iodine/ethanol solution and then with 1 g/ml starch/castor oil solution (Sigma). After drying, 50 µl of 100 µM acetylcholine (Sigma) was injected subcutaneously into the paws of mice. Pictures of the mouse footpads were taken after 5 min.

### Cell treatment

The epidermal cells transfected with foxc1 lentivirus were collected when confluent cells grow up to 85–90% and injected into the paw pad of burn mice with Microliter^TM^ Syringes (Hamilton, 7655-01). The mice were then killed 28 days later, their feet were cut, and fixed with 10% formalin overnight for frozen sections and histological analysis.

### RNA extraction and qPCR

Total RNA was prepared with Trizol. Total RNA was reverse-transcribed with TaKaRa PrimeScript^TM^ RT-PCR Kit and amplified with SYBR Premix Ex Taq^TM^ II kit (TaKaRa) according to the manufacturer’s instructions. All primers were listed in Table S1. The PCR was performed with a Eco (Illumina) system under the following procedure: initiation for 30 s at 95 °C, followed by 40 thermal cycles each at 95 °C for 3 s, 55 ℃ for 30 s, and 72 °C for 30 s, and then 95 °C for 15 s, 65 °C for 60 s, and 95 °C for 15 s. All data were analyzed with the C(t) value comparison method.

## Results

### Screening for sweat gland-inducing factors

**Fig. 1:**
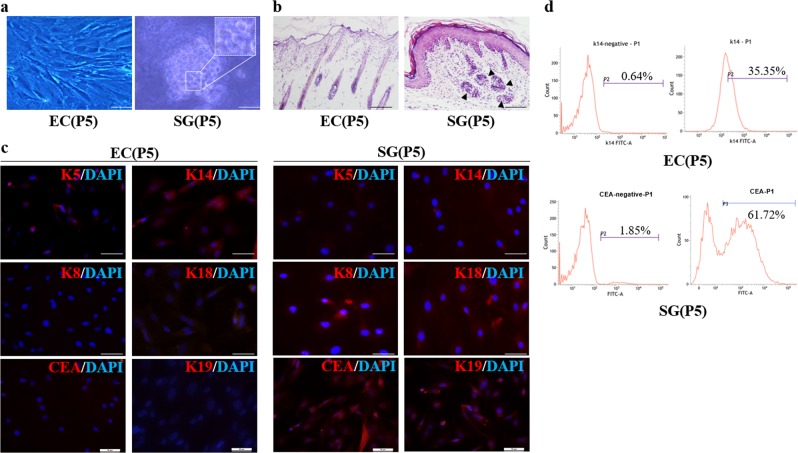
The difference between ECs and SGs in mice at P5. **a** The cell morphology of ECs and SGCs in a light microscope (scale bars: 50 μm). **b** Representative HE images of dorsal and plantar tissue. **c** Immunofluorescent staining of K5, K14, K8, K18, K19, and CEA in ECs and SGCs (K5, K14, K8, K18: red; cell nucleus: blue; scale bars: 50 μm). **d** Isolation of ECs and SGCs with the K14 antibody and CEA antibody through flow cytometry

**Fig. 2:**
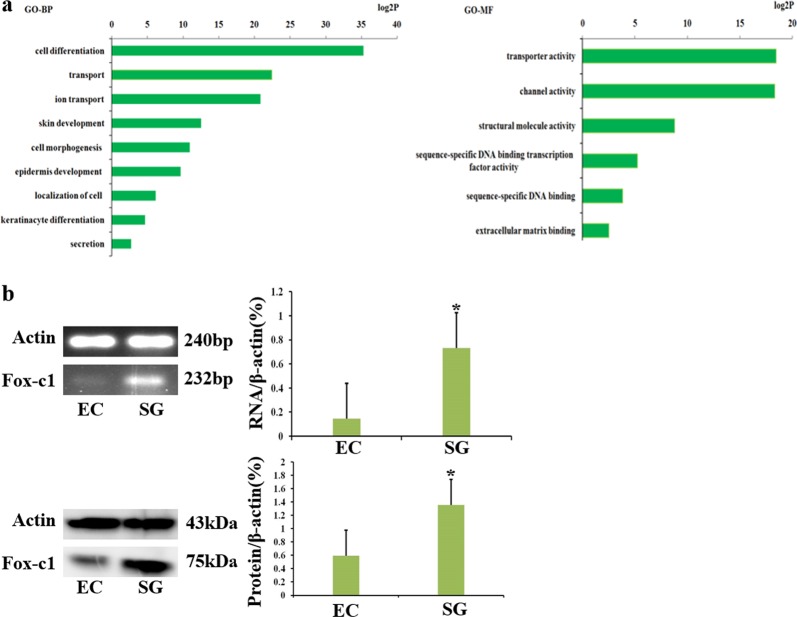
Screen SGC-specific transcriptional factors. **a** GO analysis of differential expression genes. **b** The expression of Foxc1 in ECs and SGCs at mRNA and protein level and quantification

According to the previous research on sweat gland (SG) development, we chose epidermal and sweat gland cells at postpartum 5d (P5) when they expressed significantly different gene signatures. The stratified epidermis, spindle-like cell morphology, rapid proliferation, and K5K14 expression means that epidermal cells retain stemness, while single-duct and short-coiled secretion part, clustered cells slow down cycling, and K8, K18, K19, and CEA expression shows sweat gland specification (Fig. 1). We isolated epidermal and sweat gland cells through flow cytometry with K14 and CEA antibody for transcriptome analysis. These GO categories encompassed genes involved in ion transport (e.g., *ATP1b1, Slc32a1,* and *Hcn1*), secretion (e.g., *Adcyap1, syt16,* and *tmem79*), and cell morphogenesis (e.g., *Iqub, Prdm8, Ttll1, Wt1,* and *Bmp2*), revealing that a complement of genes critical for SG-cell function and formation were activated (Fig. 2a). We identified 32 TFs (Fig. S2) that were upregulated in the specification of SG fate and selected a subset of three TFs (*FoxC1, Irf6,* and *Tfcp2l1*) on the basis of their known roles during epidermis development or their ability to enhance gland morphogenesis when expressed in the ectoderm for further functional analysis^17–22^.

### Single TF infection can induce cell viability change and sweat gland marker expression

We found that FoxC1, Irf6, and Tfcp2l1 were each capable of significantly inducing K18 expression (*P* < 0.01, normalized to control). FoxC1 upregulated the expression of most of SG-related genes significantly than SGCs (*P* < 0.05), which implicated that single FoxC1 expression drove functional sweat gland differentiation (Fig. S3)^23^. The expression of Foxc1 in ECs and SGs was verified through RT-PCR and western blotting (Fig. 2b).

**Fig. 3:**
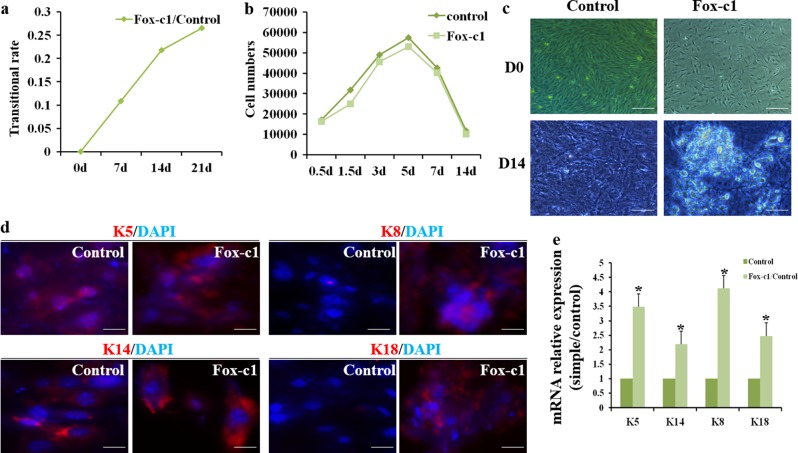
Sweat gland characteristics were observed in transfected cells. **a** Transition ratio and **b** proliferative capacity of transfected cells. **c** Morphological changes of transfected cells and control at D0 and D14 (scale bars: 200 μm). **d** Translational level of specific markers was detected by immunofluorescence staining (K5, K14, K8, K18: red; cell nucleus: blue; scale bars: 50 μm). **e** Transcriptional level of epidermal and sweat gland-specific markers was detected by qPCR

We examined the transitional rate and cell proliferation and found that the ratio of SG-like cells is increased with inductive duration and FoxC1 overexpression decreased the cell numbers relative to control (Fig. 3a, b). At the same time, they underwent changes in cell morphology, becoming bright with a clustered appearance after 7–14-day induction (Fig. 3c). Interestingly, the morphology of FoxC1-infected K8+ cells was altered, such that several cells exhibited a stick-like morphology compared with the ellipse appearance of native SGCs.

Following infection, ECs were maintained for 14 days before RNA extraction or immunocytochemistry. For SGC and EC identification, we selected K5, K14, K8, and K18 as biomarkers, respectively, according to our previous research^23^. As expected, K8 mRNA was highly enriched in induced SGCs, by 4.2-fold relative to scramble-infected ECs, whereas K18 mRNA enrichment was 2.6-fold higher (*n* = 3–5) (Fig. 3d–e). Interestingly, FoxC1 induced a significantly higher expression of K8 mRNA than native SGCs (*P* < 0.05) (Fig. S3). To control for the effect of media on induction of these markers, we performed parallel experiments in both F12 and SG-specific media and found that gene expression changes were similar regardless of growth conditions (data not shown).

Taken together, these data indicate that FoxC1 has a strong effect on SGC marker mRNA, protein expression, and cell proliferation.

### Foxc1-induced sweat gland cells are capable of sweating commitment in vitro

**Fig. 4:**
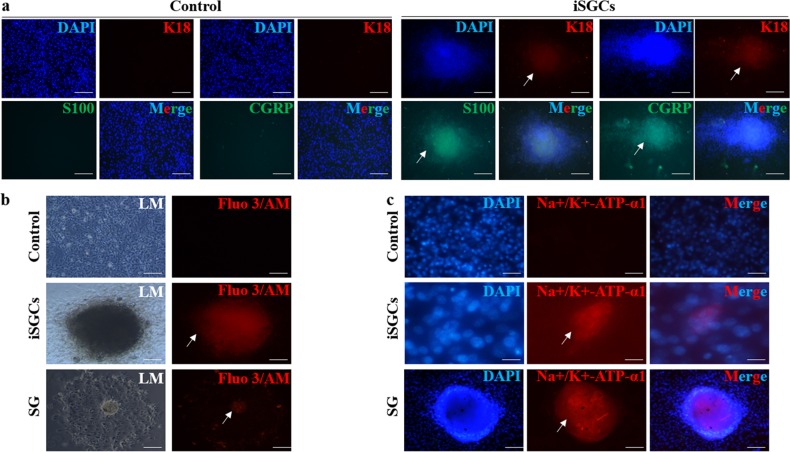
Functional analysis of induced SGCs (iSGCs) derived from ECs. **a** The coexpression of SG-specific marker K18 and dark cell-specific marker CGRP and clear cell-specific marker S100 in iSGCs and control (S100, CGRP: green; K18: red; cell nucleus: blue; scale bars: 200 μm). **b**, **c** Sweat gland functions were detected by **b** free Ca^2+^ concentration (scale bars: 200 μm) and **c** Na–K–ATPase (Ca^2+^, Na–K–ATPase: red; cell nucleus: blue; scale bars: 50 μm). (The fluorescence of cells was pointed with arrows)

The secretory portion of the sweat gland contains “clear” and “dark” secretory cells and supportive myoepithelial cells^24^. Dark and clear cells are both necessary to sweat, consistent with interdependency of them; ion channels in clear cells are ineffective in generating sweat in the abnormal dark cells^25^. To determine whether FoxC1-infected cells could obtain functional change, we performed immunostaining with S100-clear cell markers, and the calcitonin gene-related peptide (CGRP), highly expressed in the dark cells. On day 14, part of infected ECs differentiated into S100+ clear cells and CGRP+ dark cells (*n* = 3). In comparison, few control ECs were S100+ and CGRP+ (Fig. 4a).

The dynamic process of sweat secretion is thought to involve two primary forms of transport: Ca^2+^ -dependent transport and Na–KCl cotransport^25^. Ca^2+^ influx pathway is critical for CaCC (Ca^2+^-activated chloride channel) function, sweat secretion, and thermoregulation^12^. To detect the potential secretion of sweat in the infected cells, we used Fluo 3/AM to image the free intracellular Ca^2+^ levels. Ach increased the intracellular Ca^2+^ concentrations, which was reflected by an increase in the fluorescence intensity. This was observed in both the Foxc1 group and primary SG cells (Fig. 4b).

Although not a specific marker of SGCs, Na^+^–K^+^–ATPase is required for Na+, Cl+, and water transport^25^ and is highly expressed by all sweat gland lineage cells but hardly in ECs. As such, we tested Na^+^–K^+^–ATPase to determine whether FoxC1 overexpression activates a gene profile related to sweating. As expected, FoxC1 overexpression induced very strong Na^+^–K^+^–ATPase protein expression. These results suggested that the induced sweat gland cells (iSGCs) are functional and recapitulate some of the properties of sweat glands in vitro (Fig. 4c).

### Foxc1 overexpression induces a sweat gland-like gene expression signature

**Fig. 5:**
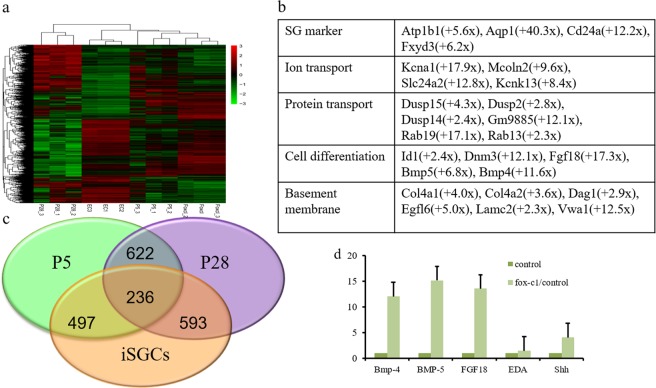
Gene expression profile. **a** Heatmap image of microarray data. Green indicates decreased expression and red indicates increased expression. **b** Functional annotations of upregulated genes in iSGCs. **c** Venn diagram of the genes in iSGCs, SG of P5 and P28. **d** mRNA expression levels in ECs and iSGCs were determined by qRT-PCR. All quantitative data were obtained from three independent experiments and are presented as mean ± SD

We next sought to determine whether TF overexpression would regulate SG-specific gene expression on a genome-wide scale. We performed microarray analysis for iSGCs and compared their expression with controls and naive SGCs (*n* = 3). We noted that the expression profiles of iSGCs were distinct from the profiles of control and similar to SGCs (Fig. 5a). TFs reduced epidermal-specific gene expression, including keratin84, Msx2, and Cdh3. Sweat gland-expressed transcripts, such as *AQP1, Atp1b1*, and *Id1*, were upregulated after FoxC1 overexpression (Fig. 5b). Among FoxC1-induced genes, we found a very significant overrepresentation of genes involved in ion transport (top GO enrichment analysis, *P* = 5.99 × 10^–10^). To determine whether FoxC1 overexpression induced SG-specific gene expression, we directly compared the enrichment of induced genes among the profile of naive SGCs (Fig. 5c). We found that FoxC1-induced genes were enriched among native SGCs relative to ECs, and that this enrichment was highly significant (*P* = 2.1 × 10^–4^).

By cell component annotation, we also found an upregulation of a subset of TFs related to SG development, including *Wnt10a, Bmp4, Eda, Shh*, and *NF-kB*^15,26^. We used quantitative real-time reverse transcriptase-PCR (qRT-PCR) to validate changes in these genes and confirmed significant upregulation (Fig. 5d).

### ISGCs recover sweat gland impairment

**Fig. 6:**
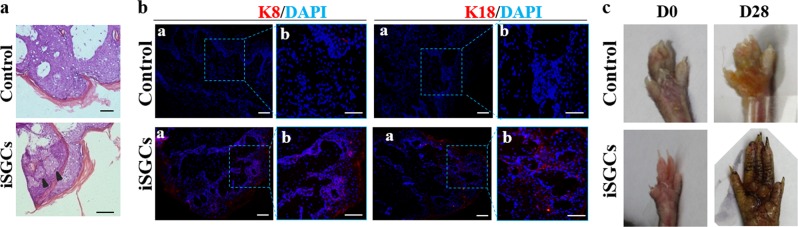
Directed regeneration of SG after transplantation of iSGCs. **a** Histology of the plantar region without treatment and transplantation of iSGCs (scale bars: 200 μm). **b** SG-specific markers K8 and K18 detected in regenerated SG tissue or control (K8, K18: red; cell nucleus: blue; scale bars: 200 and 50 μm). **c** Sweat test of mice treated with control or iSGCs

To determine the repair capacity of iSGCs in vivo, we injected 1 × 10^6^ infected ECs into the paw pad of burn mice. ECs were infected with either FoxC1 or control at 48 h before implantation. At 4 weeks post implantation, we assessed the effect of iSGCs on sweat gland regeneration. HE shows that foreign clustered cells were observed in the impaired tissue of mice transplanted with iSGCs, while the control was still unrepaired (Fig. 6a). Importantly, iSGC treatment resulted in an almost 10-fold increase in the expression of biomarkers of the secretion coil: K8 and K18 (mean ± SEM, 10 ± 0.4% *P* < 0.05, *n* = 3) (Fig. 6b) compared with the matched control. In addition, we noted a profound difference in the sweating test between iSGCs and control in the paw pad (Fig. 6c). These data indicate that iSGCs could regenerate impaired sweat gland tissue and recover sweating in vivo.

### Inhibited BMP5 expression abolishes sweat gland differentiation

**Fig. 7:**
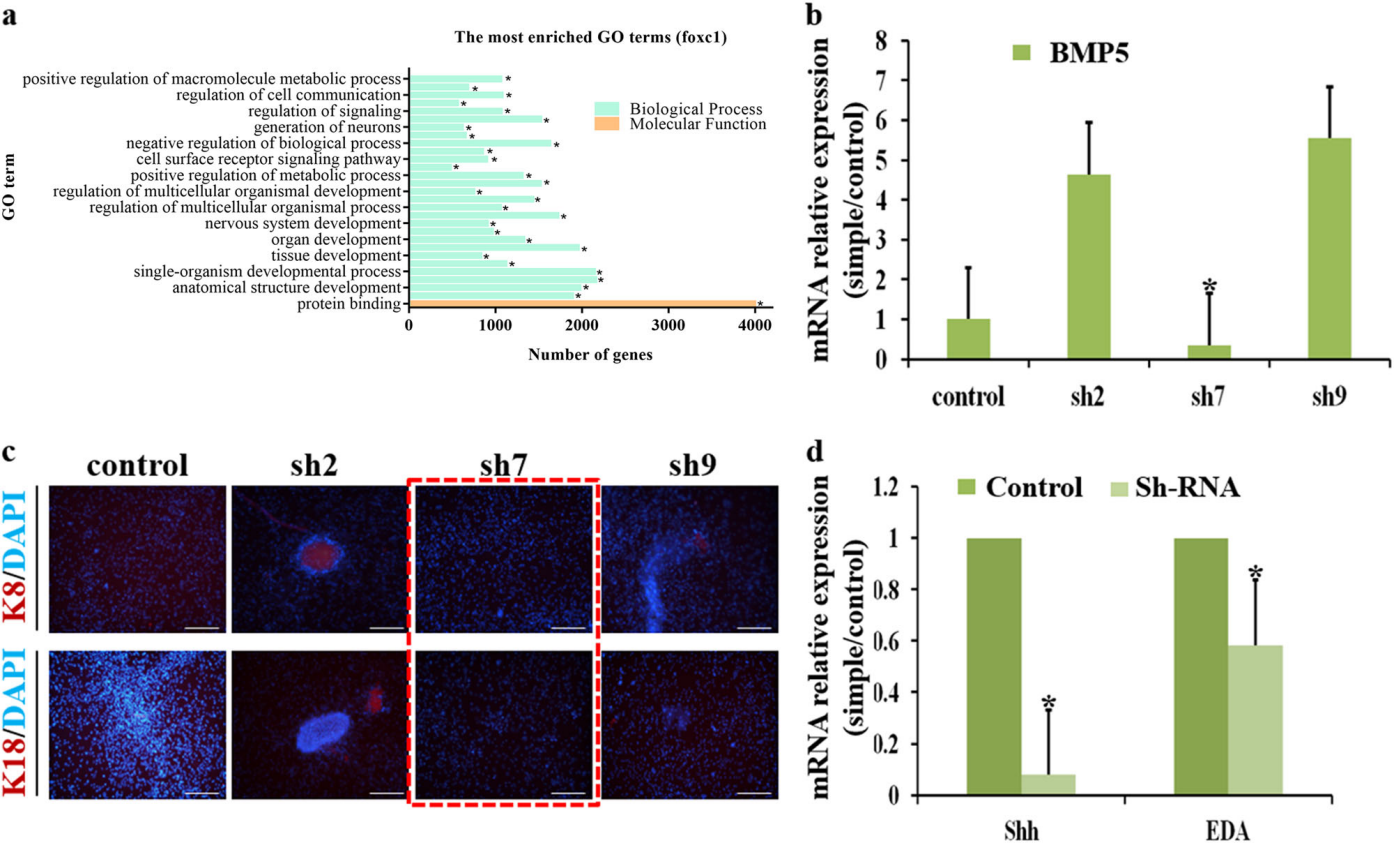
BMP5 is responsible for SG reprogramming. **a** Functional analysis of Foxc1 binding genes. **b** Inhibition of shRNA on expression of BMP5. **c** Expression of K8 and K18 in control and shRNA- transfected iSGCs (K8, K18: red; cell nucleus: blue; scale bars: 200 μm). **d** Transcriptional analysis of EDA and Shh in control and shRNA- transfected iSGCs

According to previous studies^14,26^, BMP4, BMP5, and FGF18 induced initiated SG budding and *Wnt/Eda/Shh* signaling pathway was activated during SG development. We analyzed a Foxc1 ChIP-Seq dataset and identified BMP5 likely to be targeted by Foxc1 (Fig. 7a and Table S2). Consistent with the microarray results that the expression of BMP5 was the highest in iSGCs, we suggested that Foxc1 has an active action on BMP5. To examine the functional relevance of BMP5 in SG induction, we designed three independent shRNAs against BMP5, one of which led to an 80% reduction in BMP5 levels (Fig. 7b). Analysis of BMP5 knockdown cells revealed significantly reduced expression of the SG signature genes K8 and K18 (Fig. 7c). Furthermore, compared to cells transduced with control shRNA, BMP5 knockdown cells showed reduced Eda and Shh levels (Fig. 7d). Our findings demonstrate that induction of SG fate determinants requires expression of BMP5 and provides direct evidence that Foxc1 initiated SG differentiation through activating the Wnt/Eda/Shh cascade.

## Discussion

In this study, we sought to identify TFs that may regulate SGC fate. We hypothesized that TF upregulation of inductive factors would coincide with acquisition of SGC specification. We chose K8K18 because they represent a specific and functional marker of SGCs and K5K14 are known as EC markers^23^. As both ECs and SGCs were derived from the epidermal progenitors and specified in different directions, the genetic difference is minimal and gene expression change is notably significant. The reason we picked up P5 mice is that the tip of the sweat gland duct began to coil and the morphological and functional developmental driver continuously worked until fully mature at 3 weeks after birth^26^. They show significant differences in cell shape, tissue morphology, and cytokeratin expression. Thus, genomic analysis of SGCs relative to ECs identified TFs expressed by SGCs at the time of specification. As expected, we identified several previously unidentified SGC-expressed TFs.

We found that Tfcp2l1 overexpression induced ion transport in *ATP1b1, ATP2a2,* and *Fxyd2*, suggesting that it may facilitate SGC fate; however, by itself, it was not sufficient to permit sweat gland differentiation. Interestingly, ectopic Tfcp2l1-HERVH is known to drive human embryonic stem cell pluripotency, modulating long noncoding RNAs and thereby promoting self-renewal^27^. Thus, we assumed that it may induce EC dedifferentiation to epidermal stem cells first and then re-differentiation to SGCs. Irf6 is upregulated in SGCs compared with ECs, and its expression is maintained in adult skin. In addition, Irf6 is highly expressed during both skin and oral epithelium, as well as knockout of irf6 downregulates *K14* and *Sfn* expression in early embryonic development, implicating it in the specification and development of skin, limb, and craniofacial^20,21^. Fox family binds as monomers to an asymmetric target sequence and are most likely to activate gene expression and relate to skin development^28,29^. For example, hair development in mammals involves Foxq1 and Foxn1, and mutations in Foxq1 cause aberrant differentiation of the hair shaft^30^, whereas Foxn1 leads to the nude phenotype in newborn mice^31^. Foxc1 maintains hair follicle stem cell niche and reinforces quiescence in self-renewing hair follicle stem cells^17,18^. The mice with Foxc1 ablated in skin were severely hypohidrotic for excess keratotic plug formation in sweat duct luminal cells^19^. In our research, it shows the quantitative increase in FoxC1 mRNAs in SGCs, suggesting that it may have potential to determine sweat gland fate in vitro.

Somatic cell reprogramming through various combinations of TF overexpression has been established as a novel means to induce transdifferentiation of various somatic cell types, typically fibroblasts, to pluripotent stem cells and other lineages^8^. It appears that multiple TFs play vital roles in inducing SG development from epidermal precursors, including Eda, Shh, Wnt10, Foxa1, NF-kB, and others^15^; however, the instructive role of TFs toward SGC fate has barely concerned in vitro. For example, Eda1 treatment is capable of rescuing sweat gland development in the offspring of pregnant Tabby mice^32^, but it is unknown whether Eda expression could induce SGC specification in cultured cells. Recent studies have demonstrated that NF-kB and Lef-1 overexpression in a marine fibroblast leads to sweat gland conversion, but it is restricted by the long duration and low transition^11^. As such, the rapid induction functional SGC by TF overexpression has not yet been achieved. We sought to define SGC fate through a combination of marker expression, genome-wide expression profiles, and functional characteristics. Of the factors tested, only FoxC1 overexpression was capable of inducing a proliferative sweat gland-like cell that resembles SG and recovers sweating both in vitro and in vivo.

The iSGCs generated by FoxC1 spread faster and aggregated clustered without a certain shape. iSGCs also expressed specific genes of clear cells—S100 and dark cells—CGRP, demonstrating that FoxC1 is capable of inducing functional cells differentiation. According to previous studies, we performed calcium ion flux assay on the physiological functionality of SG with Ach stimulation^33,34,35^. Although the muscarinic activator pilocarpine is specifically binding to SGCs, our previous results with pilocarpine did not show a significant difference compared with Ach stimulation (data not shown). Interestingly, although the expression of Na^+^–K^+^–ATPase in iSGCs is less than naive SGCs, their Ca^2+^ uptake capability is much stronger. It is possible that attached cells which lost their structure and less cell–cell contact would impair their physiological sensitivity and functionality. In vivo injection assay showed that iSGCs involve and promote sweat gland regeneration. HE sections represented that impaired regions were filled with newly immigrated cells, which may be transplanted cells or activated endogenous stem cells. More importantly, immunofluorescence and sweating tests showed afunctional sweat gland and partial sweating. Collectively, our data show that iSGCs function in vitro and in vivo to generate a sweat gland or may activate intrinsic repair.

The mechanisms that FoxC1 determined SG fate remain to be fully elucidated. Ectopic FoxC1 expression in cultured ECs induced *BMP5, Wnt10a, NF-kB, Shh,* and *Eda* transcription. Of upregulated genes, *BMP5* was higher followed by *Eda, NF-kB,* and *Shh*. According to previous studies, *BMP4, BMP5,* and *FGF18* synergistically initiated sweat gland development^14^. The *BMP5* shRNA infection is capable of reverse FoxC1-induced SGC fate. However, FoxC1 expression during sweat gland development was in the downstream of Shh^33^. Thus, we proposed that FoxC1 may drive sweat gland specification via a positive feedback regulation by activating *BMP5* transcription to activate the signal cascade.

As with most current reprogramming strategies, the efficiency of generating iSGCs is low, not to mention in vivo wound repair. In comparison to previous methods to derive SGCs from fibroblasts or MSCs, the direct inductive methods described here have advantages in saving time and materials. We demonstrated that overexpression of FoxC1 in ECs resulted in an increase in the efficiency of generating functional iSGCs and is a promising resource because they are easy to expand for transplantation-based studies that require large numbers of cells.

In summary, we found that directly targeting FoxC1 expression in ECs promoted SGC specification and sweat gland regeneration in burn mice. These data suggest that FoxC1 is one of the principa gatekeepers for SGC lineage fate and reveals the underlying molecular mechanism. In future, small molecules or compounds that induce FoxC1 expression might be screened to generate an affluent resource of transplanted iSGCs or enhance SGC specification directly in the wound region. From a translational perspective, our results may provide a promising strategy for clinical anhidrotic and hypohidrotic cure.

## Supplementary information

Supplementary materials
