# Supplementary material for: Direct reprogramming of epidermal cells toward sweat gland-like cells by defined factors
Source: Cell Death Dis. 2019 Mar 20;10(4):272. doi: 10.1038/s41419-019-1503-7 (PMC6426881; doi:10.1038/s41419-019-1503-7)
Supplement: Supplementary file 1 — Supplementary materials [file 41419_2019_1503_MOESM1_ESM.docx]

**Figures**

**Supplement Figure 1**


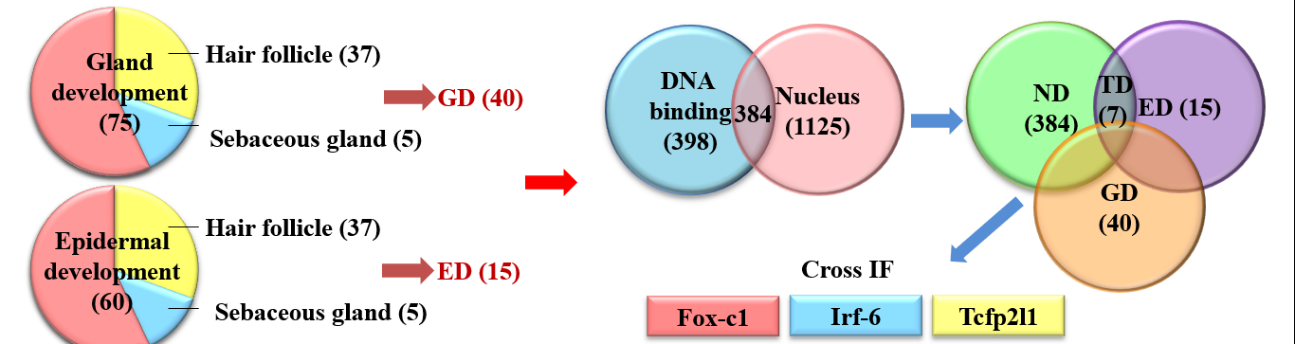


**Supplement Figure 2**


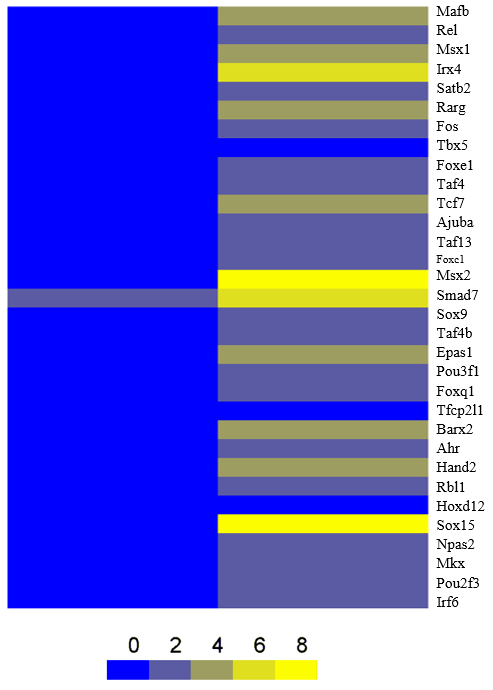


**
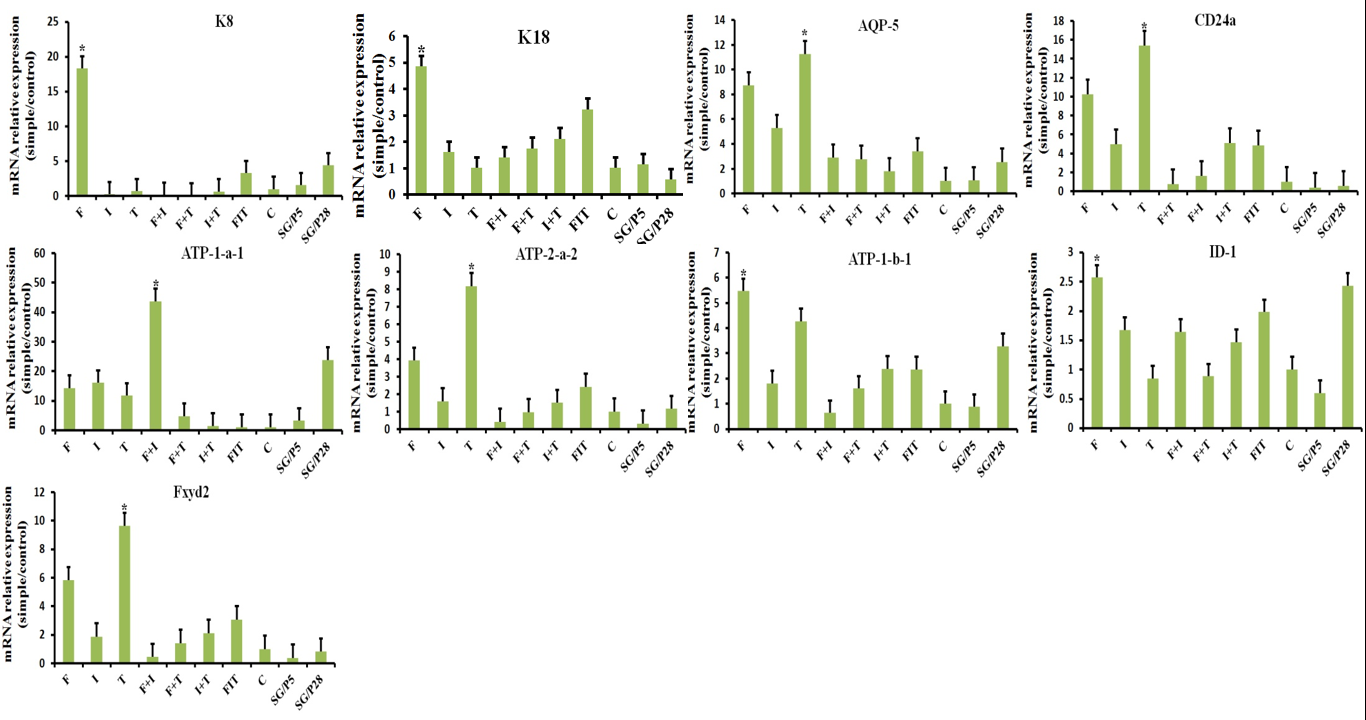
Supplement Figure 3**

**Figure legend**

**Supplement Fig. 1** Venn diagram of the progress of RNA-data analysis and transcriptional factor screen.

**Supplement Fig. 2** The heatmap of all of upregulated transcriptional factors**.**

**Supplement Fig. 3** The expression of SG related genes after transfection.

**Tables**

**Supplement Table 1**

| **Primers** | **Sequence(5'to3')** |
| --- | --- |
| **Actin-F** | **GTGGGCCGCTCTAGGCACCA** |
| **Actin-R** | **TGGCCTTAGGGTTCAGGGGGG** |
| **K5-F** | **TCCAGTGTGTCCTTCCGAAGT** |
| **K5-R** | **TGCCTCCGCCAGAACTGTA** |
| **K14-F** | **TCTTGGCGGTGGTATTGGTGAT** |
| **K14-R** | **CAGGCTCTGCTCCGTCTCAAACT** |
| **K8-F** | **GGAGGACTGAGTTCATCCTACGGG** |
| **K8-R** | **GGGTTTCAATCTTCTTCACGACCA** |
| **K18-F** | **CCGTCTTGCCGCTGATGACTTT** |
| **K18-R** | **TCCGCCATGATCTTGCTGAGGT** |
| **EDA-F** | **CCTGTTGACTGGCTTATGGTGA** |
| **EDA-R** | **AGGATACATACTTGGCAGGTTATT** |
| **Shh-F** | **GCCGATATGAAGGGAAGATCACAAGA** |
| **Shh-R** | **TCATCCCAGCCCTCAGTCACTCG** |
| **Foxc1-F** | **ACGCAGTGAAGGACAAGGAGGAGAAGG** |
| **Foxc1-R** | **ACGCTGAAGCCCTGGCTGTGATGC** |
| **ATP1a1-F** | **GACGCCTTTCAGAATGCCTACC** |
| **ATP1a1-R** | **TGTGACCATGATGACCTTAATCCC** |
| **ATP1b1-F** | **CAAACCTAAGCCTCCCAAGAAT** |
| **ATP1b1-R** | **TCTCACCATACGCCTTACACTCA** |
| **ATP2a2-F** | **AGTCTGCCTTCTGTGGAAACCC** |
| **ATP2a2-R** | **TGCTAACTCTACAAGCCCGTCAT** |
| **Fxyd2-F** | **ATGGACAGGTGGTACTTGGGTG** |
| **Fxyd2-R** | **CATTGACCTGCCTATGCTTCTTACT** |
| **Aqp5-F** | **GCCTTATCCATTGGCTTGTCTGTC** |
| **Aqp5-R** | **CCCAGTCCTCCTCCGGCTCATA** |
| **ID1-F** | **GTTCGCTGAAGGCAGGCAGGAC** |
| **ID1-R** | **CACCTTGCTCACTTTGCGGTTC** |
| **CD24a-F** | **AAATCGGCTGTTACCATTACCT** |
| **CD24a-R** | **TCCCTCTACATCAAACCTACGC** |
| **BMP4-F** | **ACTGCCGTCGCCATTCGCTCTA** |
| **BMP4-R** | **CAACACCACCTTGTCGTACTCGTC** |
| **BMP5-F** | **ACAGAGGTTGGAAGACTGCGTAA** |
| **BMP5-R** | **GACGGTTCATTGAGGTAAGGTTGT** |
| **FGF18-F** | **AAGGAGTGCGTGTTCATTGAGAAG** |
| **FGF18-R** | **CGGGATCGCTTAGTAACTGTGGT** |

**Supplement Table 2**

| **Foxc1 targeted genes** |
| --- |
| Sox17 |
| Fam150a |
| St18 |
| Pcmtd1 |
| Sntg1 |
| 3110035E14Rik |
| Mybl1 |
| Vcpip1 |
| Sgk3 |
| Arfgef1 |
| Cpa6 |
| Prex2 |
| A830018L16Rik |
| Sulf1 |
| Slco5a1 |
| Prdm14 |
| Lactb2 |
| Xkr9 |
| Eya1 |
| Msc |
| Trpa1 |
| Sbspon |
| Rdh10 |
| Stau2 |
| Gdap1 |
| Crispld1 |
| Crisp4 |
| Defb41 |
| Tfap2b |
| Pkhd1 |
| Mcm3 |
| Efhc1 |
| Tram2 |
| Kcnq5 |
| Rims1 |
| 4933415F23Rik |
| Ogfrl1 |
| B3gat2 |
| Sdhaf4 |
| Col9a1 |
| Col19a1 |
| Lmbrd1 |
| Adgrb3 |
| 4931408C20Rik |
| Phf3 |
| Lgsn |
| Bend6 |
| Dst |
| Fam168b |
| Plekhb2 |
| Hs6st1 |
| Uggt1 |
| Fer1l5 |
| Cnnm3 |
| Ankrd39 |
| Cnga3 |
| Mgat4a |
| Rev1 |
| Aff3 |
| Lonrf2 |
| Tbc1d8 |
| Rfx8 |
| Map4k4 |
| Il1r1 |
| Slc9a4 |
| Slc9a2 |
| Mfsd9 |
| Pou3f3 |
| Mrps9 |
| Fhl2 |
| Nck2 |
| 1500015O10Rik |
| Tpp2 |
| Ercc5 |
| Mettl21e |
| Gulp1 |
| Col3a1 |
| Col5a2 |
| Wdr75 |
| Slc40a1 |
| Slc39a10 |
| Tmeff2 |
| Sdpr |
| Nabp1 |
| Myo1b |
| Stat4 |
| Stat1 |
| Gls |
| Tmem194b |
| Mfsd6 |
| Inpp1 |
| 1700019D03Rik |
| Mstn |
| Stk17b |
| Hecw2 |
| Gtf3c3 |
| Pgap1 |
| Ankrd44 |
| Mob4 |
| Plcl1 |
| Hsfy2 |
| Spats2l |
| Aox1 |
| Aox4 |
| Aox2 |
| Clk1 |
| Fam126b |
| Ndufb3 |
| Cflar |
| Mpp4 |
| Als2 |
| Cdk15 |
| Fzd7 |
| Gm973 |
| Bmpr2 |
| Fam117b |
| Carf |
| Abi2 |
| Raph1 |
| Cd28 |
| Icos |
| Pard3b |
| Nrp2 |
| Ino80d |
| Ndufs1 |
| Gpr1 |
| Adam23 |
| Klf7 |
| Creb1 |
| Mettl21a |
| Ccnyl1 |
| Fzd5 |
| Plekhm3 |
| Idh1 |
| Pth2r |
| Map2 |
| Unc80 |
| Kansl1l |
| Myl1 |
| Lancl1 |
| Erbb4 |
| Ikzf2 |
| Spag16 |
| Bard1 |
| Abca12 |
| Atic |
| Fn1 |
| Mreg |
| Smarcal1 |
| Rpl37a |
| Igfbp2 |
| Igfbp5 |
| Tnp1 |
| Rufy4 |
| Arpc2 |
| Gpbar1 |
| Tmbim1 |
| Ctdsp1 |
| Nhej1 |
| Atg9a |
| Tuba4a |
| Dnpep |
| Gmppa |
| Obsl1 |
| Epha4 |
| Pax3 |
| Sgpp2 |
| Acsl3 |
| Utp14b |
| Kcne4 |
| Scg2 |
| Ap1s3 |
| Mrpl44 |
| Serpine2 |
| Fam124b |
| Cul3 |
| Dock10 |
| Nyap2 |
| Gm9747 |
| Irs1 |
| Rhbdd1 |
| Tm4sf20 |
| Sphkap |
| Pid1 |
| Dner |
| Nmur1 |
| 1700019O17Rik |
| Ptma |
| Atg16l1 |
| Sag |
| Glrp1 |
| Arl4c |
| Sh3bp4 |
| Agap1 |
| Gbx2 |
| Asb18 |
| Iqca |
| Ackr3 |
| Cops8 |
| Col6a3 |
| Mlph |
| Rab17 |
| Lrrfip1 |
| Ramp1 |
| Per2 |
| Asb1 |
| Twist2 |
| Hdac4 |
| Ndufa10 |
| Olfr1412 |
| Otos |
| Agxt |
| Crocc2 |
| Sned1 |
| Stk25 |
| D2hgdh |
| Pdcd1 |
| Fam174a |
| St8sia4 |
| D1Ertd622e |
| Ppip5k2 |
| Pam |
| Rnf152 |
| Zcchc2 |
| Phlpp1 |
| Bcl2 |
| Cdh7 |
| Cdh19 |
| Dsel |
| Cntnap5a |
| Clasp1 |
| Gli2 |
| Inhbb |
| Ptpn4 |
| C1ql2 |
| Marco |
| En1 |
| Insig2 |
| Actr3 |
| Gpr39 |
| Nckap5 |
| Mgat5 |
| Tmem163 |
| Acmsd |
| Ccnt2 |
| Mcm6 |
| Dars |
| Thsd7b |
| Cd55 |
| Zp3r |
| Il10 |
| Slc41a1 |
| Slc45a3 |
| Elk4 |
| Mfsd4 |
| Cdk18 |
| Klhdc8a |
| Dstyk |
| Cntn2 |
| Mdm4 |
| Pik3c2b |
| Ppp1r15b |
| Plekha6 |
| Sox13 |
| Lax1 |
| Btg2 |
| Chit1 |
| Tmem183a |
| Adipor1 |
| Kdm5b |
| Ube2t |
| Gpr37l1 |
| Elf3 |
| Ipo9 |
| Nav1 |
| Csrp1 |
| Mroh3 |
| Camsap2 |
| Kif14 |
| Zfp281 |
| Nr5a2 |
| Atp6v1g3 |
| Nek7 |
| Aspm |
| Cfhr1 |
| Kcnt2 |
| B3galt2 |
| Cdc73 |
| Rgs2 |
| Rgs13 |
| Rgs1 |
| Rgs18 |
| Brinp3 |
| Pla2g4a |
| Prg4 |
| Hmcn1 |
| Rnf2 |
| Fam129a |
| Edem3 |
| 1700025G04Rik |
| Colgalt2 |
| Ncf2 |
| Nmnat2 |
| Lamc2 |
| Lamc1 |
| Npl |
| Rgs8 |
| Rnasel |
| Rgsl1 |
| Cacna1e |
| Ier5 |
| Stx6 |
| BC034090 |
| Xpr1 |
| Qsox1 |
| Tor1aip1 |
| Fam163a |
| Tdrd5 |
| Axdnd1 |
| Tor3a |
| Fam20b |
| Angptl1 |
| Ralgps2 |
| Tex35 |
| Sec16b |
| Brinp2 |
| Pappa2 |
| Cacybp |
| Rabgap1l |
| Rc3h1 |
| Zbtb37 |
| Cenpl |
| Prdx6 |
| Tnfsf4 |
| Tnfsf18 |
| Suco |
| Pigc |
| Myoc |
| Prrx1 |
| Gorab |
| Mettl11b |
| Kifap3 |
| Scyl3 |
| Ccdc181 |
| Dpt |
| Xcl1 |
| Gpr161 |
| Mpc2 |
| Mpzl1 |
| Rcsd1 |
| Pou2f1 |
| Dusp27 |
| Mael |
| Ildr2 |
| Tada1 |
| Pogk |
| Gm4846 |
| Fam78b |
| Lmx1a |
| Pbx1 |
| Rgs5 |
| Rgs4 |
| 1700084C01Rik |
| Ddr2 |
| 1700015E13Rik |
| Olfml2b |
| Fcgr3 |
| Pcp4l1 |
| Ppox |
| Ufc1 |
| Dedd |
| Copa |
| Pex19 |
| Dcaf8 |
| Slamf9 |
| Cfap45 |
| Dusp23 |
| Crp |
| Aim2 |
| Cadm3 |
| Olfr427 |
| Olfr231 |
| Fmn2 |
| Grem2 |
| Rgs7 |
| Fh1 |
| Rbm8a2 |
| Sdccag8 |
| Akt3 |
| Zbtb18 |
| 1700016C15Rik |
| Efcab2 |
| Kif26b |
| Smyd3 |
| Cnst |
| Sccpdh |
| Ahctf1 |
| Cdc42bpa |
| Adck3 |
| Psen2 |
| Itpkb |
| 6330403A02Rik |
| Parp1 |
| Acbd3 |
| H3f3a |
| Tmem63a |
| Wdr26 |
| Ccdc121 |
| Enah |
| Srp9 |
| Fbxo28 |
| Capn2 |
| Ccdc185 |
| Susd4 |
| Tlr5 |
| 1700056E22Rik |
| Dusp10 |
| Hlx |
| 42795 |
| Lyplal1 |
| Tgfb2 |
| Rrp15 |
| D1Pas1 |
| Gpatch2 |
| Esrrg |
| Ush2a |
| Kctd3 |
| Cenpf |
| Ptpn14 |
| Smyd2 |
| Prox1 |
| Rps6kc1 |
| Angel2 |
| Mfsd7b |
| Tmem206 |
| Ppp2r5a |
| Slc30a1 |
| Kcnh1 |
| Hhat |
| Sertad4 |
| A130010J15Rik |
| Camk1g |
| Plxna2 |
| Cd34 |
| Cr1l |
| Cnksr3 |
| Oprm1 |
| Gm10945 |
| Rgs17 |
| Fbxo5 |
| Vip |
| Myct1 |
| Esr1 |
| Armt1 |
| Zbtb2 |
| Akap12 |
| Plekhg1 |
| Iyd |
| Tab2 |
| Ust |
| Sash1 |
| Gm9930 |
| Samd5 |
| Stxbp5 |
| Adgb |
| Grm1 |
| Fbxo30 |
| Epm2a |
| Gm9797 |
| Utrn |
| Plagl1 |
| Zc2hc1b |
| Ltv1 |
| Phactr2 |
| Adat2 |
| Aig1 |
| Hivep2 |
| Cited2 |
| Txlnb |
| Reps1 |
| Ect2l |
| Ccdc28a |
| Nhsl1 |
| Hebp2 |
| Perp |
| Tnfaip3 |
| Olig3 |
| Ifngr1 |
| Il22ra2 |
| Il20ra |
| Map3k5 |
| Map7 |
| Mtfr2 |
| Pde7b |
| Ahi1 |
| Aldh8a1 |
| 1700020N01Rik |
| Sgk1 |
| 4930444G20Rik |
| Slc2a12 |
| Tbpl1 |
| Tcf21 |
| Eya4 |
| Vnn1 |
| Taar3 |
| Taar4 |
| Moxd1 |
| Ctgf |
| Enpp1 |
| Enpp3 |
| Med23 |
| Arg1 |
| Akap7 |
| Epb41l2 |
| Gm9767 |
| Samd3 |
| L3mbtl3 |
| Lama2 |
| Ptprk |
| Themis |
| Gm9996 |
| 9330159F19Rik |
| Echdc1 |
| Rspo3 |
| Cenpw |
| Ncoa7 |
| Hddc2 |
| Tpd52l1 |
| Rnf217 |
| Nkain2 |
| Zufsp |
| Rwdd1 |
| Trappc3l |
| Fam26e |
| Fam26f |
| Dse |
| Tspyl1 |
| Tspyl4 |
| Frk |
| Amd2 |
| Hdac2 |
| Marcks |
| Lama4 |
| Wisp3 |
| Fyn |
| Traf3ip2 |
| G630090E17Rik |
| Slc16a10 |
| Rpf2 |
| Amd1 |
| Cdk19 |
| Slc22a16 |
| Mettl24 |
| Wasf1 |
| Zbtb24 |
| Cd164 |
| Ccdc162 |
| Cep57l1 |
| Sesn1 |
| Armc2 |
| Snx3 |
| Nr2e1 |
| Sec63 |
| Scml4 |
| Sobp |
| Pdss2 |
| F930017D23Rik |
| Rtn4ip1 |
| Atg5 |
| Prdm1 |
| Lin28b |
| Grik2 |
| Ascc3 |
| Sim1 |
| Fam162b |
| Gprc6a |
| 4933411G06Rik |
| Vgll2 |
| Ros1 |
| Pln |
| Cep85l |
| Asf1a |
| Fam184a |
| Man1a |
| Msl3l2 |
| Tbc1d32 |
| Gja1 |
| Gm9956 |
| Serinc1 |
| Pkib |
| Smpdl3a |
| Lims1 |
| Ccdc138 |
| Edar |
| Sh3rf3 |
| 42988 |
| P4ha1 |
| Oit3 |
| Gm10322 |
| Micu1 |
| Dnajb12 |
| Ddit4 |
| Anapc16 |
| Spock2 |
| 4632428N05Rik |
| Cdh23 |
| Unc5b |
| Sgpl1 |
| Adamts14 |
| Prf1 |
| Pald1 |
| Eif4ebp2 |
| Lrrc20 |
| D830039M14Rik |
| Npffr1 |
| H2afy2 |
| Neurog3 |
| Tspan15 |
| Tacr2 |
| Hk1 |
| Hkdc1 |
| Kif1bp |
| Ddx21 |
| Ccar1 |
| Mypn |
| Dnajc12 |
| Ctnna3 |
| Gm10118 |
| Reep3 |
| Jmjd1c |
| Egr2 |
| Gm10797 |
| Rtkn2 |
| Arid5b |
| 1700040L02Rik |
| Tmem26 |
| Rhobtb1 |
| Cdk1 |
| Ank3 |
| Ccdc6 |
| Fam13c |
| 4930533K18Rik |
| Bicc1 |
| Ipmk |
| Gm9923 |
| Zwint |
| Pcdh15 |
| Gnaz |
| Bcr |
| Specc1l |
| Adora2a |
| Upb1 |
| Cabin1 |
| Mif |
| Slc5a4b |
| Dip2a |
| Col6a1 |
| Pcbp3 |
| Col18a1 |
| Gm10941 |
| Adarb1 |
| Pfkl |
| Ilvbl |
| C2cd4c |
| Lppr3 |
| Cnn2 |
| Stk11 |
| Btbd2 |
| Gadd45b |
| Slc39a3 |
| Pias4 |
| Dapk3 |
| BC025920 |
| Glt8d2 |
| 1500009L16Rik |
| Nuak1 |
| Tcp11l2 |
| Rfx4 |
| Ric8b |
| Mterf2 |
| Btbd11 |
| Pwp1 |
| Ascl4 |
| Rtcb |
| Fbxo7 |
| Timp3 |
| Syn3 |
| Hsp90b1 |
| Ttc41 |
| Fabp3-ps1 |
| Nt5dc3 |
| Pah |
| Igf1 |
| Parpbp |
| Gnptab |
| Ano4 |
| Gas2l3 |
| Tmpo |
| Nedd1 |
| Elk3 |
| Hal |
| Ntn4 |
| Fgd6 |
| Nr2c1 |
| Tmcc3 |
| Cep83 |
| Plxnc1 |
| Cradd |
| Socs2 |
| Mrpl42 |
| Anapc15-ps |
| Btg1 |
| Dcn |
| Lum |
| Kera |
| Epyc |
| Gm10754 |
| Atp2b1 |
| Poc1b |
| Dusp6 |
| Csl |
| Kitl |
| 1700017N19Rik |
| Mgat4c |
| Rassf9 |
| Alx1 |
| Tmtc2 |
| Ppfia2 |
| Acss3 |
| Lin7a |
| Myf5 |
| Ptprq |
| Ppp1r12a |
| Syt1 |
| Nav3 |
| E2f7 |
| Csrp2 |
| Osbpl8 |
| Nap1l1 |
| Phlda1 |
| Krr1 |
| Kcnc2 |
| Atxn7l3b |
| Trhde |
| Tph2 |
| Tbc1d15 |
| Zfc3h1 |
| Lgr5 |
| Tspan8 |
| Ptprr |
| 4933416C03Rik |
| Myrfl |
| Best3 |
| Cct2 |
| Frs2 |
| Cpsf6 |
| Cpm |
| Mdm2 |
| Slc35e3 |
| Rap1b |
| Mdm1 |
| Ifng |
| Cand1 |
| Tmbim4 |
| Llph |
| Hmga2 |
| Wif1 |
| Tbc1d30 |
| Rassf3 |
| D930020B18Rik |
| Srgap1 |
| Tmem5 |
| Avpr1a |
| Ppm1h |
| Mon2 |
| Usp15 |
| Fam19a2 |
| Slc16a7 |
| Lrig3 |
| Xrcc6bp1 |
| Os9 |
| Gli1 |
| R3hdm2 |
| Lrp1 |
| Nab2 |
| Tmem194 |
| Myo1a |
| Spryd4 |
| Timeless |
| Slc39a5 |
| Myl6b |
| Zc3h10 |
| Erbb3 |
| Rab5b |
| Olfr820 |
| Olfr822 |
| Neurod4 |
| Vmn2r84 |
| Patz1 |
| 4921536K21Rik |
| Pes1 |
| Tbc1d10a |
| Nf2 |
| Nipsnap1 |
| Emid1 |
| Znrf3 |
| Ankrd36 |
| Myl7 |
| Ykt6 |
| Zmiz2 |
| H2afv |
| Tbrg4 |
| Adcy1 |
| Igfbp3 |
| Tns3 |
| Pkd1l1 |
| Vwc2 |
| Fignl1 |
| Ddc |
| Grb10 |
| Cobl |
| Egfr |
| Ppp3r1 |
| Etaa1 |
| Meis1 |
| Spred2 |
| Actr2 |
| Cep68 |
| Sertad2 |
| Peli1 |
| Ugp2 |
| Wdpcp |
| Otx1 |
| Cct4 |
| Usp34 |
| Bcl11a |
| 5730522E02Rik |
| Fancl |
| Ccdc85a |
| Efemp1 |
| Eml6 |
| Sptbn1 |
| 4930505A04Rik |
| Gpr75 |
| Ubtd2 |
| Stk10 |
| Fgf18 |
| Fam196b |
| Slit3 |
| Tenm2 |
| Mat2b |
| Ccng1 |
| Gabrg2 |
| Gabra1 |
| Ccnjl |
| 4933415A04Rik |
| Pwwp2a |
| Ttc1 |
| Adra1b |
| Rnf145 |
| Ebf1 |
| Clint1 |
| Thg1l |
| Nipal4 |
| Fndc9 |
| Havcr2 |
| Zfp62 |
| Rnf130 |
| Sqstm1 |
| Hnrnph1 |
| Rufy1 |
| Col23a1 |
| Phykpl |
| 9530068E07Rik |
| Fstl4 |
| Aff4 |
| Rad50 |
| P4ha2 |
| Fnip1 |
| Rapgef6 |
| Gpx3 |
| Tnip1 |
| Anxa6 |
| Gria1 |
| Fam114a2 |
| Irgm2 |
| Prss38 |
| Lrrc48 |
| Alkbh5 |
| Mief2 |
| Top3a |
| Usp22 |
| Epn2 |
| Prpsap2 |
| Specc1 |
| Adora2b |
| Trim16 |
| Fbxw10 |
| Cdrt4 |
| Tekt3 |
| Pmp22 |
| Hs3st3a1 |
| Elac2 |
| Zkscan6 |
| Shisa6 |
| Pirt |
| Gas7 |
| Dhrs7c |
| Pik3r6 |
| Ccdc42 |
| Myh10 |
| Rnf222 |
| Odf4 |
| Ctc1 |
| Per1 |
| Chd3 |
| Tmem88 |
| Kdm6b |
| Trp53 |
| Atp1b2 |
| Polr2a |
| Kctd11 |
| Eif5a |
| Asgr1 |
| Asgr2 |
| 0610010K14Rik |
| Gm21988 |
| Tm4sf5 |
| Mink1 |
| Slc25a11 |
| Pfn1 |
| Spag7 |
| Camta2 |
| Rabep1 |
| Nup88 |
| Ggt6 |
| Spns2 |
| Atp2a3 |
| Shpk |
| Rap1gap2 |
| Hic1 |
| Rtn4rl1 |
| Rpa1 |
| Inpp5k |
| Doc2b |
| Rph3al |
| Fam101b |
| Vps53 |
| Rnmtl1 |
| Nxn |
| Timm22 |
| Abr |
| Cpd |
| Tmigd1 |
| Efcab5 |
| Gm10277 |
| Pipox |
| Phf12 |
| Fam222b |
| Nek8 |
| Sdf2 |
| Nos2 |
| Ksr1 |
| Wsb1 |
| Gm9964 |
| Omg |
| Rab11fip4 |
| Suz12 |
| Crlf3 |
| Rhbdl3 |
| Myo1d |
| Spaca3 |
| 5530401A14Rik |
| Ccl12 |
| Ccl8 |
| Tmem132e |
| Lig3 |
| Rffl |
| Slfn9 |
| Taf15 |
| Ccl5 |
| Ddx52 |
| Synrg |
| Dusp14 |
| Tada2a |
| Acaca |
| Aatf |
| Znhit3 |
| 1700125H20Rik |
| Ppm1d |
| Bcas3 |
| Tbx2 |
| Tbx4 |
| Med13 |
| Dhx40 |
| Ypel2 |
| Prr11 |
| Ska2 |
| Rad51c |
| Gm11492 |
| Cuedc1 |
| Mrps23 |
| Ccdc182 |
| Msi2 |
| Akap1 |
| Coil |
| Nog |
| 4932411E22Rik |
| Ankfn1 |
| Tmem100 |
| Hlf |
| Stxbp4 |
| Cox11 |
| Kif2b |
| Utp18 |
| Nme1 |
| Tob1 |
| Wfikkn2 |
| Luc7l3 |
| Ankrd40 |
| Abcc3 |
| Acsf2 |
| Sgca |
| Fam117a |
| Ngfr |
| Phb |
| Igf2bp1 |
| Hoxb13 |
| Hoxb1 |
| Skap1 |
| Mrpl45 |
| Gpr179 |
| Arhgap23 |
| Pcgf2 |
| Cwc25 |
| Tcap |
| Ikzf3 |
| Csf3 |
| Nr1d1 |
| Msl1 |
| Tns4 |
| Ccr7 |
| Krt24 |
| Krt28 |
| Krtap4-8 |
| Hspb9 |
| Stat5b |
| Stat3 |
| Ptrf |
| Plekhh3 |
| Ezh1 |
| Ramp2 |
| Coa3 |
| Becn1 |
| Nbr1 |
| Rdm1 |
| Etv4 |
| Cd300lg |
| Hdac5 |
| Gpatch8 |
| Fzd2 |
| C1ql1 |
| Hexim2 |
| Wnt3 |
| Nsf |
| Kansl1 |
| Myl4 |
| Mrc2 |
| 42804 |
| Taco1 |
| Map3k3 |
| Prr29 |
| Ern1 |
| Tex2 |
| Smurf2 |
| Bptf |
| Pitpnc1 |
| Prkca |
| Cep112 |
| Axin2 |
| E030025P04Rik |
| Gna13 |
| Arsg |
| Wipi1 |
| 1700012B07Rik |
| Abca8b |
| Abca8a |
| Abca9 |
| Map2k6 |
| Kcnj16 |
| Kcnj2 |
| Sox9 |
| Cdc42ep4 |
| Sdk2 |
| Ttyh2 |
| Gpr142 |
| Gprc5c |
| Rab37 |
| Nat9 |
| Armc7 |
| Slc25a19 |
| Grb2 |
| Caskin2 |
| H3f3b |
| Wbp2 |
| Rnf157 |
| Ubald2 |
| Rhbdf2 |
| St6galnac1 |
| Mxra7 |
| Mfsd11 |
| Mgat5b |
| Sec14l1 |
| 42987 |
| Gm11733 |
| Tnrc6c |
| Cyth1 |
| BC100451 |
| Timp2 |
| Cbx4 |
| Chmp6 |
| 1810043H04Rik |
| Bahcc1 |
| Actg1 |
| Dcxr |
| Cbr2 |
| Csnk1d |
| Zfp750 |
| Metrnl |
| Dtnb |
| Dnmt3a |
| Ncoa1 |
| Itsn2 |
| Mfsd2b |
| Klhl29 |
| Apob |
| Ldah |
| Hs1bp3 |
| Rhob |
| Sdc1 |
| Matn3 |
| Wdr35 |
| Ttc32 |
| Osr1 |
| Nt5c1b |
| Rdh14 |
| Pgk1-rs7 |
| Kcns3 |
| Vsnl1 |
| Fam49a |
| Rpl36-ps3 |
| Mycn |
| Ddx1 |
| Nbas |
| Fam84a |
| Trib2 |
| E2f6 |
| Rock2 |
| Pqlc3 |
| Kcnf1 |
| Gm2174 |
| Atp6v1c2 |
| Odc1 |
| Hpcal1 |
| Adam17 |
| Rrm2 |
| Kidins220 |
| Id2 |
| Rnf144a |
| Cmpk2 |
| Sox11 |
| Adi1 |
| Tssc1 |
| Pxdn |
| Sntg2 |
| Tmem18 |
| Fam110c |
| Lamb1 |
| Slc26a4 |
| Hbp1 |
| Prkar2b |
| Pik3cg |
| Cdhr3 |
| Atxn7l1 |
| Ferd3l |
| Twist1 |
| Hdac9 |
| 4921508M14Rik |
| Snx13 |
| Ahr |
| Agr3 |
| Agr2 |
| Tspan13 |
| Gm5434 |
| Bzw2 |
| Sostdc1 |
| Ispd |
| Meox2 |
| Agmo |
| Dgkb |
| Etv1 |
| Arl4a |
| Scin |
| Lsmem1 |
| Gm7008 |
| Ifrd1 |
| Zfp277 |
| Dock4 |
| Immp2l |
| Lrrn3 |
| Dnajb9 |
| Nrcam |
| Stxbp6 |
| Prkd1 |
| G2e3 |
| Coch |
| Hectd1 |
| Gpr33 |
| Nubpl |
| Arhgap5 |
| Akap6 |
| Npas3 |
| Egln3 |
| Sptssa |
| Snx6 |
| Nfkbia |
| Insm2 |
| Nkx2-1 |
| Nkx2-9 |
| Pax9 |
| Slc25a21 |
| 4921506M07Rik |
| Sstr1 |
| Clec14a |
| Sec23a |
| Gemin2 |
| Lrfn5 |
| Gm527 |
| Fancm |
| Wdr20rt |
| Rpl10l |
| Mdga2 |
| Klhdc2 |
| Arf6 |
| Gm9887 |
| Vcpkmt |
| Sos2 |
| Map4k5 |
| Tmx1 |
| Frmd6 |
| Arid4a |
| 2700049A03Rik |
| Dact1 |
| Daam1 |
| Gpr135 |
| Lrrc9 |
| Pcnxl4 |
| Ppm1a |
| 4930447C04Rik |
| Six6 |
| Six1 |
| Trmt5 |
| Slc38a6 |
| D830013O20Rik |
| Tmem30b |
| Prkch |
| Hif1a |
| Syt16 |
| Dbpht2 |
| Gm11042 |
| Kcnh5 |
| Rhoj |
| Gphb5 |
| Sgpp1 |
| Syne2 |
| Hspa2 |
| Gm10451 |
| Gpx2 |
| Fntb |
| Max |
| Fut8 |
| Gphn |
| Arg2 |
| Zfyve26 |
| Rad51b |
| Zfp36l1 |
| Actn1 |
| Dcaf5 |
| Plekhd1 |
| Susd6 |
| Smoc1 |
| Map3k9 |
| Sipa1l1 |
| Dcaf4 |
| 2410016O06Rik |
| Aldh6a1 |
| Ltbp2 |
| Eif2b2 |
| Mlh3 |
| Tmed10 |
| Fos |
| Jdp2 |
| Batf |
| 0610007P14Rik |
| Ttll5 |
| Tgfb3 |
| Esrrb |
| Angel1 |
| Irf2bpl |
| Tmed8 |
| Ahsa1 |
| Snw1 |
| Nrxn3 |
| Cep128 |
| Tshr |
| Gtf2a1 |
| Flrt2 |
| Galc |
| Gpr65 |
| Ptpn21 |
| Eml5 |
| Foxn3 |
| Efcab11 |
| Kcnk13 |
| Psmc1 |
| Nrde2 |
| Ttc7b |
| Rps6ka5 |
| Tc2n |
| Atxn3 |
| Cpsf2 |
| Slc24a4 |
| Rin3 |
| Lgmn |
| Chga |
| Itpk1 |
| Btbd7 |
| Unc79 |
| Prima1 |
| Ddx24 |
| Gsc |
| Gm10000 |
| Dicer1 |
| Clmn |
| 4930408O17Rik |
| Syne3 |
| Tcl1 |
| Bdkrb2 |
| Papola |
| Vrk1 |
| Bcl11b |
| Ccnk |
| Degs2 |
| Wars |
| Dlk1 |
| Rtl1 |
| Dync1h1 |
| Hsp90aa1 |
| Wdr20 |
| Ankrd9 |
| Amn |
| Cdc42bpb |
| Gm266 |
| Mark3 |
| Klc1 |
| 2010107E04Rik |
| A730018C14Rik |
| A530016L24Rik |
| Akt1 |
| Cep170b |
| BC022687 |
| Brf1 |
| Wdr60 |
| Ncapg2 |
| Rapgef5 |
| Cdca7l |
| Dnah11 |
| Sp4 |
| Sp8 |
| Abcb5 |
| Itgb8 |
| Macc1 |
| Tmem196 |
| Calml3 |
| Net1 |
| Akr1c14 |
| Akr1c13 |
| Akr1c19 |
| Gm5444 |
| Klf6 |
| Pitrm1 |
| Adarb2 |
| Wdr37 |
| Dip2c |
| Zmynd11 |
| Chrm3 |
| Ryr2 |
| Mtr |
| Nid1 |
| Lyst |
| Tbce |
| Gli3 |
| Inhba |
| Sugct |
| Mplkip |
| Rala |
| Yae1d1 |
| Vdac3-ps1 |
| Pou6f2 |
| Vps41 |
| Stard3nl |
| Sfrp4 |
| Elmo1 |
| Aoah |
| Trim27 |
| Gpx5 |
| Hist1h1b |
| Hist1h3c |
| Hist1h2an |
| Zfp184 |
| Pom121l2 |
| Btn1a1 |
| Hist1h4h |
| Hist1h2af |
| Hist1h3e |
| Hist1h2bg |
| Hist1h4d |
| Hist1h2be |
| Hist1h4c |
| Hfe |
| Hist1h2bb |
| Hist1h3a |
| Hist1h1a |
| Slc17a1 |
| Lrrc16a |
| Fam65b |
| Acot13 |
| Dcdc2a |
| Nrsn1 |
| Hdgfl1 |
| Prl |
| Prl3c1 |
| Prl8a2 |
| Prl5a1 |
| Cdkal1 |
| Uqcrfs1 |
| Hus1b |
| Gm5447 |
| Foxq1 |
| Foxf2 |
| Foxc1 |
| Gmds |
| Mylk4 |
| Serpinb9 |
| Tubb2b |
| Psmg4 |
| Slc22a23 |
| Pxdc1 |
| Fam50b |
| Prpf4b |
| Cdyl |
| Rpp40 |
| Fars2 |
| Nrn1 |
| Ly86 |
| Rreb1 |
| Ssr1 |
| Riok1 |
| Dsp |
| Bmp6 |
| Bloc1s5 |
| Slc35b3 |
| Ofcc1 |
| Tfap2a |
| Gcnt2 |
| Mak |
| Elovl2 |
| Nedd9 |
| Tmem170b |
| Gm5082 |
| Adtrp |
| Hivep1 |
| Edn1 |
| Phactr1 |
| Gfod1 |
| Ranbp9 |
| Rnf182 |
| Cd83 |
| Jarid2 |
| Dtnbp1 |
| Mylip |
| Atxn1 |
| Gm10113 |
| Fam8a1 |
| Rnf144b |
| Id4 |
| Ptpdc1 |
| Barx1 |
| Phf2 |
| Fam120a |
| Ninj1 |
| Bicd2 |
| Aspn |
| Omd |
| Ogn |
| Spin1 |
| Nxnl2 |
| S1pr3 |
| Shc3 |
| Sema4d |
| Gadd45g |
| Diras2 |
| Syk |
| Auh |
| Nfil3 |
| Ror2 |
| Msx2 |
| Gm5449 |
| Drd1 |
| Cplx2 |
| Gprin1 |
| Nsd1 |
| Ddx46 |
| B230219D22Rik |
| Pitx1 |
| H2afy |
| Lect2 |
| Tgfbi |
| Smad5 |
| Spock1 |
| Idnk |
| Slc28a3 |
| Ntrk2 |
| Agtpbp1 |
| Naa35 |
| Zcchc6 |
| Gas1 |
| Gm5084 |
| Dapk1 |
| Fbp2 |
| Fbp1 |
| 2010111I01Rik |
| Ptch1 |
| Ercc6l2 |
| Hsd17b3 |
| Habp4 |
| Aaed1 |
| Cdk20 |
| Hiatl1 |
| Rsl1 |
| Zfp456 |
| Zfp874b |
| Adcy2 |
| Papd7 |
| Srd5a1 |
| Ube2ql1 |
| Ice1 |
| Adamts16 |
| 8030423J24Rik |
| Irx1 |
| Irx2 |
| Irx4 |
| Clptm1l |
| Tert |
| Cast |
| Pcsk1 |
| Ell2 |
| Glrx |
| Rfesd |
| Gpr150 |
| Ttc37 |
| Slf1 |
| 2210408I21Rik |
| Fam172a |
| Nr2f1 |
| Arrdc3 |
| Adgrv1 |
| Lysmd3 |
| Cetn3 |
| Mef2c |
| Tmem161b |
| Ccnh |
| Rasa1 |
| Cox7c |
| Edil3 |
| Hapln1 |
| Vcan |
| Atp6ap1l |
| Rps23 |
| Atg10 |
| Ssbp2 |
| Rasgrf2 |
| Ankrd34b |
| Spz1 |
| Serinc5 |
| Thbs4 |
| Cmya5 |
| Papd4 |
| Jmy |
| Arsb |
| Lhfpl2 |
| Ap3b1 |
| Tbca |
| Otp |
| Wdr41 |
| Zbed3 |
| Crhbp |
| S100z |
| F2rl1 |
| F2r |
| F2rl2 |
| Iqgap2 |
| Sv2c |
| Poc5 |
| Ankdd1b |
| Polk |
| Col4a3bp |
| 1700029F12Rik |
| Fam169a |
| Enc1 |
| Gm10260 |
| Arhgef28 |
| Foxd1 |
| Fcho2 |
| Tnpo1 |
| Ptcd2 |
| Map1b |
| Cartpt |
| Slc30a5 |
| Pik3r1 |
| Cd180 |
| Mast4 |
| Srek1 |
| Erbb2ip |
| Nln |
| Adamts6 |
| Fam159b |
| Rgs7bp |
| 4933425L06Rik |
| Htr1a |
| 3830408C21Rik |
| Apoo-ps |
| Zswim6 |
| Smim15 |
| Ndufaf2 |
| Ercc8 |
| Depdc1b |
| Pde4d |
| Rab3c |
| Plk2 |
| Actbl2 |
| Map3k1 |
| Ankrd55 |
| Il6st |
| Slc38a9 |
| Ppap2a |
| Ccno |
| Cdc20b |
| Esm1 |
| Snx18 |
| Arl15 |
| Ndufs4 |
| Fst |
| Mocs2 |
| Itga2 |
| Isl1 |
| Parp8 |
| Emb |
| Hcn1 |
| Mrps30 |
| Nnt |
| Flnb |
| Dnase1l3 |
| Pxk |
| Oit1 |
| 4930452B06Rik |
| Fhit |
| Ptprg |
| 3830406C13Rik |
| Cadps |
| Synpr |
| Sntn |
| Atxn7 |
| Slc4a7 |
| Ngly1 |
| Oxsm |
| Top2b |
| Rarb |
| Nid2 |
| Gng2 |
| Saysd1 |
| Usp54 |
| Sec24c |
| Fut11 |
| Vcl |
| Kat6b |
| Dupd1 |
| Comtd1 |
| Zfp503 |
| 1700112E06Rik |
| 1700112E06Rik |
| Kcnma1 |
| Dlg5 |
| E330034G19Rik |
| Zmiz1 |
| Arf4 |
| Il17rd |
| Arhgef3 |
| Fam208a |
| Lrtm1 |
| Cacna2d3 |
| Actr8 |
| Chdh |
| Cacna1d |
| Tkt |
| Sfmbt1 |
| Tmem110 |
| Itih4 |
| Smim4 |
| Stab1 |
| Sh3bp5 |
| Colq |
| Btd |
| Ankrd28 |
| Ercc6 |
| 1810011H11Rik |
| Lrrc18 |
| Wdfy4 |
| Arhgap22 |
| Mapk8 |
| Gdf10 |
| Rbp3 |
| Gm5460 |
| Npy4r |
| Fam35a |
| Bmpr1a |
| Opn4 |
| Ccser2 |
| Ghitm |
| Nrg3 |
| Sh2d4b |
| Mbl1 |
| Ptgdr |
| Fermt2 |
| Gm15217 |
| Bmp4 |
| Cdkn3 |
| Fbxo34 |
| Ktn1 |
| Peli2 |
| Tmem260 |
| Otx2 |
| Naa30 |
| 1700011H14Rik |
| 3632451O06Rik |
| Olfr726 |
| Rnase12 |
| Olfr750 |
| Rnase4 |
| Eddm3b |
| Rnase2a |
| Vmn2r89 |
| Mettl17 |
| Ndrg2 |
| Hnrnpc |
| Mettl3 |
| Trav21-dv12 |
| Trdv4 |
| Traj37 |
| Trac |
| Abhd4 |
| Oxa1l |
| Slc7a7 |
| Prmt5 |
| Ajuba |
| 4931414P19Rik |
| Ngdn |
| Dhrs2 |
| Dhrs4 |
| Pck2 |
| Dcaf11 |
| Psme2 |
| Nfatc4 |
| Nynrin |
| Cbln3 |
| Sdr39u1 |
| Gzmb |
| Gja3 |
| Ift88 |
| N6amt2 |
| Lats2 |
| Sap18 |
| Zdhhc20 |
| Micu2 |
| Fgf9 |
| Rpl13-ps3 |
| 1700129C05Rik |
| Cab39l |
| Cdadc1 |
| Shisa2 |
| Amer2 |
| Spata13 |
| C1qtnf9 |
| Mipep |
| Tnfrsf19 |
| Sacs |
| Sgcg |
| Kpna3 |
| Dleu7 |
| Rnaseh2b |
| Gucy1b2 |
| Fam124a |
| Ctsb |
| Fdft1 |
| Gata4 |
| Fam167a |
| Pinx1 |
| Sox7 |
| Rp1l1 |
| Prss55 |
| Msra |
| Hmbox1 |
| Ints9 |
| Extl3 |
| Fzd3 |
| Fbxo16 |
| Zfp395 |
| Pnoc |
| Elp3 |
| Scara5 |
| Ccdc25 |
| Gulo |
| Stmn4 |
| Gm10032 |
| Bnip3l |
| Ppp2r2a |
| Ebf2 |
| Cdca2 |
| Dock5 |
| Nefl |
| Nefm |
| Adam7 |
| Adam28 |
| Stc1 |
| Pebp4 |
| Egr3 |
| Ppp3cc |
| Phyhip |
| Hr |
| Xpo7 |
| Dok2 |
| Gfra2 |
| Fndc3a |
| Cysltr2 |
| Rcbtb2 |
| Lpar6 |
| Rb1 |
| Itm2b |
| Med4 |
| Nudt15 |
| Htr2a |
| Esd |
| Lrch1 |
| 5031414D18Rik |
| Spert |
| Erich6b |
| Cog3 |
| Slc25a30 |
| Kctd4 |
| Nufip1 |
| Tsc22d1 |
| Serp2 |
| Lacc1 |
| Enox1 |
| Gm1587 |
| Dnajc15 |
| Fam216b |
| Dgkh |
| Vwa8 |
| Zfp957 |
| Mtrf1 |
| Wbp4 |
| Sugt1 |
| Lect1 |
| Olfm4 |
| Pcdh17 |
| Diap3 |
| Tdrd3 |
| Pcdh20 |
| Gm10110 |
| Pcdh9 |
| 4921530L21Rik |
| Dach1 |
| Mzt1 |
| Klf5 |
| Klf12 |
| Prr30 |
| Tbc1d4 |
| Lmo7 |
| Scel |
| Slain1 |
| Ednrb |
| Rnf219 |
| Rbm26 |
| Gm10076 |
| Spry2 |
| Trim52 |
| Slitrk1 |
| Slitrk6 |
| Slitrk5 |
| Gpc5 |
| Gpc6 |
| Dct |
| Gpr180 |
| Abcc4 |
| Dnajc3 |
| Hs6st3 |
| Oxgr1 |
| Mbnl2 |
| Rap2a |
| Ipo5 |
| Farp1 |
| Stk24 |
| Slc15a1 |
| Dock9 |
| Gpr183 |
| Gm5089 |
| Pcca |
| Ggact |
| Tmtc4 |
| Nalcn |
| Fgf14 |
| Sepp1 |
| Ghr |
| Plcxd3 |
| Prkaa1 |
| Ptger4 |
| Dab2 |
| Fyb |
| Rictor |
| Osmr |
| Lifr |
| Egflam |
| Gdnf |
| Wdr70 |
| 2410089E03Rik |
| Slc1a3 |
| Ranbp3l |
| Nadk2 |
| Ugt3a1 |
| Prlr |
| Ttc23l |
| Rai14 |
| C1qtnf3 |
| Adamts12 |
| Tars |
| Npr3 |
| Zfr |
| Mtmr12 |
| Golph3 |
| Pdzd2 |
| Cdh6 |
| Cdh9 |
| Cdh10 |
| Cdh12 |
| Cdh18 |
| 9230109A22Rik |
| Gm5468 |
| Myo10 |
| Fam134b |
| Zfp622 |
| 42805 |
| Fbxl7 |
| Ank |
| Otulin |
| Fam105a |
| Trio |
| Dnah5 |
| Ctnnd2 |
| Dap |
| 42800 |
| Cmbl |
| Fam173b |
| Tas2r119 |
| Sema5a |
| Sdc2 |
| Cpq |
| Nipal2 |
| Kcns2 |
| Stk3 |
| Osr2 |
| Vps13b |
| Cox6c |
| Rgs22 |
| Rnf19a |
| Rpl7a-ps3 |
| Ankrd46 |
| Ywhaz |
| Gm10384 |
| Grhl2 |
| Rrm2b |
| Odf1 |
| Azin1 |
| Atp6v1c1 |
| Baalc |
| Fzd6 |
| Dcstamp |
| Dpys |
| Lrp12 |
| Zfpm2 |
| Oxr1 |
| Abra |
| Angpt1 |
| Rspo2 |
| Emc2 |
| Tmem74 |
| Trhr |
| Csmd3 |
| Trps1 |
| Eif3h |
| Utp23 |
| Rad21 |
| Aard |
| Slc30a8 |
| Med30 |
| Ext1 |
| Gm7489 |
| Samd12 |
| Colec10 |
| Nov |
| Enpp2 |
| Col14a1 |
| Mrpl13 |
| Mtbp |
| Sntb1 |
| Has2 |
| Slc22a22 |
| Zhx2 |
| Derl1 |
| Gm29394 |
| Klhl38 |
| Fer1l6 |
| Trmt12 |
| Ndufb9 |
| Mtss1 |
| Nsmce2 |
| Trib1 |
| A1bg |
| Myc |
| Gsdmc |
| Gsdmc4 |
| Fam49b |
| Asap1 |
| Adcy8 |
| Hhla1 |
| Tmem71 |
| Phf20l1 |
| Tg |
| Ndrg1 |
| St3gal1 |
| Zfat |
| Gm10282 |
| Khdrbs3 |
| Fam135b |
| Col22a1 |
| Trappc9 |
| Dennd3 |
| Slc45a4 |
| Adgrb1 |
| Cyp11b1 |
| Gsdmd |
| Tsta3 |
| Puf60 |
| BC024139 |
| Plec |
| Spatc1 |
| Oplah |
| 1110038F14Rik |
| Rbfox2 |
| Apol7a |
| Apol8 |
| Ncf4 |
| Rac2 |
| Mfng |
| Gga1 |
| Triobp |
| Micall1 |
| Pla2g6 |
| Kcnj4 |
| Josd1 |
| Rpl3 |
| Tab1 |
| Mgat3 |
| Tnrc6b |
| Sgsm3 |
| Slc25a17 |
| Zc3h7b |
| Tef |
| 1500009C09Rik |
| Fam109b |
| Ndufa6 |
| Tcf20 |
| Nfam1 |
| Serhl |
| Cyb5r3 |
| Scube1 |
| Parvb |
| Parvg |
| 1810041L15Rik |
| Prr5 |
| Phf21b |
| Nup50 |
| Ribc2 |
| Fbln1 |
| Wnt7b |
| Cerk |
| Tbc1d22a |
| Fam19a5 |
| Zdhhc25 |
| Pim3 |
| Ttll8 |
| Mov10l1 |
| Selo |
| Plxnb2 |
| Tymp |
| Cpne8 |
| Kif21a |
| Abcd2 |
| CN725425 |
| Lrrk2 |
| Pdzrn4 |
| Pphln1 |
| Prickle1 |
| Adamts20 |
| Pus7l |
| Twf1 |
| Tmem117 |
| Dbx2 |
| Ano6 |
| Arid2 |
| Scaf11 |
| Slc38a1 |
| Slc38a2 |
| Slc38a4 |
| Amigo2 |
| Pced1b |
| Rpap3 |
| Endou |
| Hdac7 |
| Col2a1 |
| H1fnt |
| Rnd1 |
| Fkbp11 |
| Prph |
| Tmbim6 |
| Nckap5l |
| Bcdin3d |
| Racgap1 |
| Gpd1 |
| Lima1 |
| Larp4 |
| Dip2b |
| Mettl7a2 |
| Slc4a8 |
| Scn8a |
| Ankrd33 |
| Acvrl1 |
| Acvr1b |
| Grasp |
| Nr4a1 |
| Krt7 |
| Krt75 |
| Krt74 |
| Tns2 |
| Soat2 |
| Itgb7 |
| Rarg |
| Sp7 |
| Sp1 |
| Gm10337 |
| Gm28047 |
| Atp5g2 |
| Cbx5 |
| Nfe2 |
| Copz1 |
| Gpr84 |
| Itga5 |
| Ppp1r1a |
| Zfp263 |
| Adcy9 |
| Tfap4 |
| Vasn |
| Ubald1 |
| Rbfox1 |
| Abat |
| Grin2a |
| Atf7ip2 |
| Emp2 |
| Socs1 |
| Rmi2 |
| Tnfrsf17 |
| Snx29 |
| Shisa9 |
| Ercc4 |
| Mkl2 |
| Parn |
| Pdxdc1 |
| Mpv17l |
| Snai2 |
| Efcab1 |
| Mcm4 |
| Prkdc |
| Fgd4 |
| Aifm3 |
| Klhl22 |
| Scarf2 |
| Gsc2 |
| Txnrd2 |
| Comt |
| Gnb1l |
| Gm16314 |
| Tbx1 |
| Cldn5 |
| Cdc45 |
| A930003A15Rik |
| Klhl6 |
| Klhl24 |
| Abcc5 |
| Ap2m1 |
| Ephb3 |
| Vps8 |
| 2510009E07Rik |
| Map3k13 |
| Igf2bp2 |
| Etv5 |
| Dgkg |
| Crygs |
| Tbccd1 |
| Hrg |
| St6gal1 |
| BC106179 |
| Sst |
| Bcl6 |
| Lpp |
| Tprg |
| P3h2 |
| Cldn1 |
| Cldn16 |
| Il1rap |
| Gmnc |
| Ostn |
| Ccdc50 |
| Fgf12 |
| Mb21d2 |
| Hrasls |
| Atp13a5 |
| Atp13a4 |
| Opa1 |
| Gm1968 |
| Hes1 |
| Tmem44 |
| Fam43a |
| Xxylt1 |
| Acap2 |
| Apod |
| Bdh1 |
| Dlg1 |
| Mfi2 |
| Bex6 |
| Rnf168 |
| Tfrc |
| Lrch3 |
| Zfp148 |
| Slc12a8 |
| Heg1 |
| Itgb5 |
| Kalrn |
| Ccdc14 |
| Mylk |
| Hacd2 |
| Sema5b |
| Kpna1 |
| Fam162a |
| Stxbp5l |
| Gtf2e1 |
| Rabl3 |
| Ndufb4 |
| Fstl1 |
| Pla1a |
| Arhgap31 |
| B4galt4 |
| Lsamp |
| Gap43 |
| Zbtb20 |
| Zdhhc23 |
| Gramd1c |
| Naa50 |
| Gm608 |
| Boc |
| BC027231 |
| Gtpbp8 |
| Cd200r2 |
| Cd200r3 |
| Btla |
| Cd200 |
| Gcsam |
| Abhd10 |
| Phldb2 |
| Pvrl3 |
| Dppa2 |
| Trat1 |
| Ift57 |
| Bbx |
| Ccdc54 |
| Cblb |
| Alcam |
| Zpld1 |
| Impg2 |
| Abi3bp |
| Tbc1d23 |
| Tmem30c |
| Cmss1 |
| Filip1l |
| Col8a1 |
| Dcbld2 |
| St3gal6 |
| Cpox |
| Gpr15 |
| Mina |
| Crybg3 |
| Pros1 |
| Epha3 |
| Csnka2ip |
| 4930453N24Rik |
| Cggbp1 |
| Htr1f |
| Chmp2b |
| Speer2 |
| Gbe1 |
| Robo1 |
| Robo2 |
| Lipi |
| Nrip1 |
| Gm9843 |
| Usp25 |
| Gm11146 |
| Cxadr |
| D16Ertd472e |
| Tmprss15 |
| Ncam2 |
| Jam2 |
| Gabpa |
| App |
| Cyyr1 |
| Adamts1 |
| Adamts5 |
| N6amt1 |
| Cct8 |
| Bach1 |
| Grik1 |
| Cldn8 |
| 2310079G19Rik |
| 2310061N02Rik |
| Gm10229 |
| Krtap8-1 |
| Tiam1 |
| Scaf4 |
| Hunk |
| Mis18a |
| Urb1 |
| Paxbp1 |
| Olig1 |
| Ifnar2 |
| Il10rb |
| Son |
| Cryzl1 |
| Itsn1 |
| Mrps6 |
| Kcne2 |
| 4930563D23Rik |
| Kcne1 |
| Runx1 |
| Setd4 |
| Cbr1 |
| Cbr3 |
| Chaf1b |
| Cldn14 |
| Sim2 |
| Kcnj6 |
| Kcnj15 |
| Erg |
| Ets2 |
| Sh3bgr |
| Igsf5 |
| Pcp4 |
| Dscam |
| Bace2 |
| Prdm15 |
| Scaf8 |
| Tiam2 |
| Tfb1m |
| Nox3 |
| Arid1b |
| Tmem242 |
| Synj2 |
| Serac1 |
| Ezr |
| Rps6ka2 |
| Tcp10a |
| Fndc1 |
| Mpc1 |
| T |
| 1700010I14Rik |
| Pabpc6 |
| Qk |
| Park2 |
| Agpat4 |
| Map3k4 |
| Slc22a3 |
| Igf2r |
| Mas1 |
| Dact2 |
| Smoc2 |
| Thbs2 |
| Psmb1 |
| Pdcd2 |
| Chd1 |
| Rgmb |
| Lix1 |
| Has1 |
| Vmn1r233 |
| Zfp53 |
| Zfp52 |
| Zfp760 |
| Zfp945 |
| Zfp13 |
| Prss21 |
| Pdpk1 |
| Atp6v0c |
| Nthl1 |
| Tbl3 |
| Fahd1 |
| Tmem204 |
| Lmf1 |
| Narfl |
| Axin1 |
| Fam234a |
| Neurl1b |
| Dusp1 |
| Ergic1 |
| Atp6v0e |
| Crebrf |
| Nkx2-5 |
| Uqcc2 |
| Spdef |
| Anks1 |
| Clpsl2 |
| Mapk13 |
| Cpne5 |
| Pim1 |
| Ccdc167 |
| Zfand3 |
| Btbd9 |
| Glo1 |
| Dnah8 |
| Pde9a |
| Cryaa |
| Sik1 |
| Brd4 |
| Zfp871 |
| Zfp101 |
| Adamts10 |
| 42796 |
| Pfdn6 |
| H2-Oa |
| Notch4 |
| Fkbpl |
| Atf6b |
| Vars |
| Msh5 |
| Gm16181 |
| Cdsn |
| Ier3 |
| Flot1 |
| H2-T24 |
| Trim26 |
| Trim15 |
| Mog |
| Esp1 |
| Cenpq |
| Opn5 |
| Cd2ap |
| Adgrf1 |
| Pla2g7 |
| Rcan2 |
| Clic5 |
| Supt3 |
| Cdc5l |
| Hsp90ab1 |
| Tmem63b |
| Vegfa |
| Tjap1 |
| Ttbk1 |
| Ptk7 |
| Rpl7l1 |
| A330017A19Rik |
| Prph2 |
| Taf8 |
| Ccnd3 |
| Mdfi |
| 1700067P10Rik |
| Oard1 |
| Apobec2 |
| Unc5cl |
| Mocs1 |
| Daam2 |
| Kif6 |
| Rftn1 |
| Dazl |
| Plcl2 |
| Gm7334 |
| Tbc1d5 |
| Satb1 |
| Kcnh8 |
| Pp2d1 |
| Sgol1 |
| Slc5a7 |
| St6gal2 |
| Hdgfrp2 |
| Tnfaip8l1 |
| Fem1a |
| Ptprs |
| Catsperd |
| Rfx2 |
| Gtf2f1 |
| Slc25a23 |
| Pdzph1 |
| Efna5 |
| Fbxl17 |
| A930002H24Rik |
| Fer |
| Pja2 |
| Man2a1 |
| 4930583I09Rik |
| Tmem232 |
| Vapa |
| Rab31 |
| Twsg1 |
| Mtcl1 |
| Rab12 |
| Themis3 |
| Ptprm |
| Lrrc30 |
| Lama1 |
| Arhgap28 |
| L3mbtl4 |
| Epb41l3 |
| Zbtb14 |
| Dlgap1 |
| Tgif1 |
| Myl12b |
| Myom1 |
| Lpin2 |
| Emilin2 |
| Wdr43 |
| Fam179a |
| Clip4 |
| Alk |
| Ypel5 |
| Lclat1 |
| Capn13 |
| Xdh |
| Srd5a2 |
| Memo1 |
| Spast |
| Yipf4 |
| Ltbp1 |
| Rasgrp3 |
| Crim1 |
| Fez2 |
| Gm10093 |
| Vit |
| Strn |
| Prkd3 |
| Qpct |
| Cdc42ep3 |
| 4921513D11Rik |
| Cyp1b1 |
| Atl2 |
| Gemin6 |
| Morn2 |
| Sos1 |
| Cdkl4 |
| Thumpd2 |
| Gm11096 |
| Gm6594 |
| Pkdcc |
| Eml4 |
| Cox7a2l |
| Kcng3 |
| Mta3 |
| Haao |
| Zfp36l2 |
| Plekhh2 |
| Dync2li1 |
| Abcg5 |
| Ppm1b |
| Prepl |
| Camkmt |
| Six3 |
| Six2 |
| Srbd1 |
| Gm10309 |
| Epas1 |
| Rhoq |
| Socs5 |
| Mcfd2 |
| Epcam |
| Kcnk12 |
| Fbxo11 |
| Gtf2a1l |
| Lhcgr |
| Fshr |
| Gm10184 |
| Gm6741 |
| Crem |
| Cul2 |
| Bambi |
| Lyzl1 |
| Map3k8 |
| Mtpap |
| 9430020K01Rik |
| Svil |
| Zfp438 |
| Zeb1 |
| Arhgap12 |
| Kif5b |
| Epc1 |
| Rab18 |
| Mkx |
| Armc4 |
| Mpp7 |
| Wac |
| Fzd8 |
| Gjd4 |
| Ccny |
| Colec12 |
| Usp14 |
| Greb1l |
| Gata6 |
| Rbbp8 |
| Cables1 |
| Tmem241 |
| Npc1 |
| Ankrd29 |
| Lama3 |
| Osbpl1a |
| Hrh4 |
| Zfp521 |
| Gm5160 |
| Taf4b |
| Kctd1 |
| Aqp4 |
| Chst9 |
| Cdh2 |
| Dsc1 |
| Dsg1c |
| Dsg3 |
| Dsg2 |
| Rnf125 |
| Rnf138 |
| Garem |
| Klhl14 |
| Ccdc178 |
| Asxl3 |
| Nol4 |
| Dtna |
| Ino80c |
| Pik3c3 |
| Sap130 |
| Ammecr1l |
| Ercc3 |
| Stard4 |
| Nrep |
| Gm10549 |
| Epb41l4a |
| Reep5 |
| Pkd2l2 |
| Brd8 |
| Fam53c |
| Ctnna1 |
| Lrrtm2 |
| Sil1 |
| Gm5239 |
| Slc23a1 |
| Mzb1 |
| Tmem173 |
| Cxxc5 |
| Nrg2 |
| Pfdn1 |
| Slc4a9 |
| Pcdhga8 |
| Diap1 |
| Rell2 |
| Arap3 |
| 0610009O20Rik |
| Ndfip1 |
| Spry4 |
| Fgf1 |
| Arhgap26 |
| Nr3c1 |
| Pabpc2 |
| Prelid2 |
| Lars |
| Rbm27 |
| Gpr151 |
| Ppp2r2b |
| Stk32a |
| Dpysl3 |
| Jakmip2 |
| Gm94 |
| Myot |
| Dcp2 |
| Mcc |
| Ythdc2 |
| Kcnn2 |
| Fem1c |
| Ticam2 |
| Eif1a |
| Cdo1 |
| Atg12 |
| Ap3s1 |
| Lvrn |
| Arl14epl |
| Sema6a |
| Eno1b |
| Dtwd2 |
| Dmxl1 |
| Hsd17b4 |
| Fam170a |
| Hdhd1a |
| Prr16 |
| Gm4950 |
| Ftmt |
| Srfbp1 |
| Zfp474 |
| Sncaip |
| Snx2 |
| Snx24 |
| Ppic |
| Prdm6 |
| Cep120 |
| Csnk1g3 |
| Zfp608 |
| Gramd3 |
| Tex43 |
| Lmnb1 |
| 42797 |
| C330018D20Rik |
| Megf10 |
| Prrc1 |
| Ctxn3 |
| Slc12a2 |
| Fbn2 |
| Slc27a6 |
| Adamts19 |
| A730017C20Rik |
| Chsy3 |
| Smim3 |
| Dctn4 |
| Myoz3 |
| Synpo |
| Ndst1 |
| Csnk1a1 |
| Il17b |
| Sh3tc2 |
| Adrb2 |
| Gm9949 |
| Spink13 |
| Apcdd1 |
| Napg |
| Piezo2 |
| Wdr7 |
| Onecut2 |
| Fech |
| Atp8b1 |
| Nedd4l |
| Alpk2 |
| Zfp532 |
| Oacyl |
| Rax |
| Lman1 |
| Ccbe1 |
| Mppe1 |
| Impa2 |
| Slmo1 |
| Spire1 |
| Psmg2 |
| Ptpn2 |
| Ldlrad4 |
| Fam210a |
| Mc2r |
| 4930546C10Rik |
| Tcf4 |
| Ccdc68 |
| Rab27b |
| 4930503L19Rik |
| Poli |
| Mbd2 |
| Dcc |
| Mex3c |
| Smad4 |
| Elac1 |
| Gm9925 |
| Acaa2 |
| Dym |
| Smad7 |
| Ctif |
| Zbtb7c |
| Smad2 |
| Ier3ip1 |
| Hdhd2 |
| Pias2 |
| St8sia5 |
| Rnf165 |
| Epg5 |
| Slc14a1 |
| Slc14a2 |
| Setbp1 |
| Pard6g |
| Adnp2 |
| Pqlc1 |
| Ctdp1 |
| Nfatc1 |
| Atp9b |
| Sall3 |
| Zfp236 |
| Zfp516 |
| Tshz1 |
| Zadh2 |
| Zfp407 |
| Cndp1 |
| Timm21 |
| Neto1 |
| Cbln2 |
| Rttn |
| Cd226 |
| Dok6 |
| Tmx3 |
| Ppp6r3 |
| Ndufs8 |
| Aldh3b2 |
| Gstp2 |
| Syt12 |
| 2010003K11Rik |
| Npas4 |
| Brms1 |
| Cnih2 |
| Gal3st3 |
| Pcnxl3 |
| Scyl1 |
| Dpf2 |
| Snx15 |
| Nrxn2 |
| Fermt3 |
| Otub1 |
| Rtn3 |
| Pla2g16 |
| Taf6l |
| Zbtb3 |
| Lrrn4cl |
| Scgb1a1 |
| Rab3il1 |
| Fads3 |
| Tmem258 |
| Dagla |
| Sdhaf2 |
| Tmem216 |
| Ddb1 |
| Zp1 |
| Ccdc86 |
| 1700017D01Rik |
| Mrpl16 |
| Olfr1423 |
| Olfr1424 |
| Olfr1425 |
| Mpeg1 |
| Dtx4 |
| Cntf |
| Zfp91 |
| Lpxn |
| Olfr1505 |
| Tle4 |
| Psat1 |
| Gnaq |
| Gna14 |
| Prune2 |
| Gcnt1 |
| Rfk |
| Ostf1 |
| Carnmt1 |
| D030056L22Rik |
| Rorb |
| Anxa1 |
| Aldh1a7 |
| Tmc1 |
| Zfand5 |
| Gda |
| 1110059E24Rik |
| Abhd17b |
| Tmem2 |
| Trpm3 |
| Mamdc2 |
| Apba1 |
| Fam189a2 |
| Tjp2 |
| Tmem252 |
| Pgm5 |
| Gm10053 |
| Dock8 |
| Kank1 |
| Dmrt3 |
| Dmrt2 |
| Smarca2 |
| Gm815 |
| Vldlr |
| Kcnv2 |
| Pum3 |
| Rfx3 |
| 4430402I18Rik |
| Ak3 |
| Insl6 |
| Pdcd1lg2 |
| Ranbp6 |
| Il33 |
| Cstf2t |
| Prkg1 |
| Asah2 |
| Sgms1 |
| Rpl9-ps6 |
| Papss2 |
| Atad1 |
| Pten |
| Rnls |
| Stambpl1 |
| Acta2 |
| Fas |
| Lipa |
| Ifit1 |
| Ankrd1 |
| Hectd2 |
| Ppp1r3c |
| Tnks2 |
| Fgfbp3 |
| Btaf1 |
| Cpeb3 |
| Hhex |
| Exoc6 |
| Cyp26a1 |
| Myof |
| Ffar4 |
| Fra10ac1 |
| Lgi1 |
| Plce1 |
| Noc3l |
| Tbc1d12 |
| Pdlim1 |
| Sorbs1 |
| Aldh18a1 |
| Entpd1 |
| Opalin |
| Lcor |
| Slit1 |
| Frat2 |
| Pi4k2a |
| Crtac1 |
| Hpse2 |
| Nkx2-3 |
| Entpd7 |
| Cox15 |
| Cyp2c44 |
| Scd2 |
| Wnt8b |
| Fam178a |
| Lzts2 |
| Sfxn3 |
| Kazald1 |
| Lbx1 |
| Btrc |
| Fbxw4 |
| Mgea5 |
| Kcnip2 |
| Hps6 |
| Ldb1 |
| Gbf1 |
| Nfkb2 |
| Sufu |
| Trim8 |
| Arl3 |
| Sfxn2 |
| Wbp1l |
| Cyp17a1 |
| As3mt |
| Cnnm2 |
| Nt5c2 |
| Neurl1a |
| Sh3pxd2a |
| Sfr1 |
| Cfap43 |
| Gsto2 |
| Cfap58 |
| Sorcs3 |
| Rpl13a-ps1 |
| Ins1 |
| Xpnpep1 |
| Add3 |
| Mxi1 |
| Smndc1 |
| Rbm20 |
| Pdcd4 |
| Adra2a |
| Gpam |
| Acsl5 |
| Vti1a |
| Tcf7l2 |
| Plekhs1 |
| Nhlrc2 |
| Adrb1 |
| Tdrd1 |
| Vwa2 |
| Ablim1 |
| Fam160b1 |
| Trub1 |
| Atrnl1 |
| Gfra1 |
| Ccdc172 |
| Pnlip |
| Shtn1 |
| Rps12-ps3 |
| Pdzd8 |
| Emx2 |
| Rab11fip2 |
| Prlhr |
| Cacul1 |
| Grk5 |
| Zfp950 |
| Fam171a1 |
| Nmt2 |
| Fam107b |
| Frmd4a |
| Bend7 |
| Sephs1 |
| Ccdc3 |
| Camk1d |
| Nudt5 |
| Usp6nl |
| 9230102O04Rik |
| Taf3 |
| Itih5 |
| Sfmbt2 |
| Il2ra |
| Ankrd16 |
| Itga8 |
| Fam188a |
| Pter |
| C1ql3 |
| Rsu1 |
| Cubn |
| Trdmt1 |
| Vim |
| Cacnb2 |
| Malrd1 |
| Plxdc2 |
| Nebl |
| H2afb1 |
| Skida1 |
| Mllt10 |
| Dnajc1 |
| Bmi1 |
| Pip4k2a |
| 4930426L09Rik |
| Armc3 |
| Otud1 |
| Etl4 |
| Arhgap21 |
| Myo3a |
| Gad2 |
| Acbd5 |
| Spopl |
| Hnmt |
| Il1rn |
| Pax8 |
| Cacna1b |
| Ehmt1 |
| Nsmf |
| Tor4a |
| Tubb4b |
| Man1b1 |
| Sohlh1 |
| Camsap1 |
| Qsox2 |
| Pmpca |
| Notch1 |
| Cacfd1 |
| Rxra |
| Gbgt1 |
| Barhl1 |
| 1700101E01Rik |
| Ttf1 |
| Rapgef1 |
| Gle1 |
| Ccbl1 |
| Ppp2r4 |
| Ier5l |
| Cstad |
| Ptges |
| Gpr107 |
| Hmcn2 |
| Ass1 |
| Abl1 |
| Prrc2b |
| Naif1 |
| Ak1 |
| Angptl2 |
| Ralgps1 |
| Lmx1b |
| Pbx3 |
| Hc |
| Cntrl |
| Gsn |
| Stom |
| 4930402F06Rik |
| Dab2ip |
| Ttll11 |
| Lhx6 |
| Mrrf |
| Olfr350 |
| Crb2 |
| Olfml2a |
| Arhgap15 |
| Gtdc1 |
| Zeb2 |
| Orc4 |
| Mbd5 |
| Epc2 |
| Kif5c |
| Lypd6b |
| Lypd6 |
| Mmadhc |
| Rnd3 |
| Tas2r134 |
| Rbm43 |
| Rif1 |
| Fmnl2 |
| Prpf40a |
| Galnt13 |
| Kcnj3 |
| Gpd2 |
| Galnt5 |
| Acvr1c |
| Acvr1 |
| Upp2 |
| Pkp4 |
| Dapl1 |
| Tanc1 |
| 42801 |
| Cd302 |
| Itgb6 |
| Tank |
| Tbr1 |
| Slc4a10 |
| Kcnh7 |
| Fign |
| Grb14 |
| Cobll1 |
| Scn3a |
| Scn2a1 |
| Csrnp3 |
| Galnt3 |
| Scn9a |
| Xirp2 |
| B3galt1 |
| Stk39 |
| 4933409G03Rik |
| Cers6 |
| Nostrin |
| Spc25 |
| Dhrs9 |
| Lrp2 |
| Ubr3 |
| Gorasp2 |
| Tlk1 |
| Mettl8 |
| Gm17374 |
| Dync1i2 |
| Slc25a12 |
| Metap1d |
| Dlx1 |
| Dlx2 |
| Itga6 |
| Rapgef4 |
| Zak |
| Cdca7 |
| Sp9 |
| Wipf1 |
| Chn1 |
| Atf2 |
| Atp5g3 |
| Evx2 |
| Hoxd13 |
| Mtx2 |
| Ttc30b |
| Ttc30a1 |
| Osbpl6 |
| Prkra |
| Ttn |
| Ccdc141 |
| Ube2e3 |
| Itga4 |
| Cerkl |
| Neurod1 |
| Ssfa2 |
| Ppp1r1c |
| Pde1a |
| Dnajc10 |
| Frzb |
| Fsip2 |
| Zc3h15 |
| Itgav |
| Calcrl |
| Tfpi |
| Ypel4 |
| Slc43a3 |
| Tnks1bp1 |
| Aplnr |
| Olfr987 |
| Olfr1136 |
| Fnbp4 |
| Arfgap2 |
| Lrp4 |
| Ambra1 |
| Phf21a |
| Cry2 |
| Tspan18 |
| Gm10803 |
| Alx4 |
| Accs |
| Hsd17b12 |
| Api5 |
| Lrrc4c |
| Gm10801 |
| Gm10800 |
| Rag2 |
| Rag1 |
| Prr5l |
| Commd9 |
| Fjx1 |
| Pamr1 |
| Slc1a2 |
| Cd44 |
| Pdhx |
| Apip |
| Cat |
| Abtb2 |
| Nat10 |
| A930018P22Rik |
| Hipk3 |
| Prrg4 |
| Wt1 |
| Them7 |
| Rcn1 |
| Elp4 |
| Mpped2 |
| Arl14ep |
| Ccdc34 |
| Bbox1 |
| Muc15 |
| Ano3 |
| Lpcat4 |
| Ryr3 |
| Tmco5b |
| Grem1 |
| Scg5 |
| Arhgap11a |
| Actc1 |
| Dph6 |
| Meis2 |
| Spred1 |
| Rasgrp1 |
| Thbs1 |
| Fsip1 |
| Srp14 |
| Bmf |
| Pak6 |
| Rad51 |
| Rmdn3 |
| Dll4 |
| Chac1 |
| Chp1 |
| Mapkbp1 |
| Sptbn5 |
| Vps39 |
| Epb42 |
| Frmd5 |
| Eif3j1 |
| Sord |
| Shf |
| Gatm |
| Sqrdl |
| Sema6d |
| Slc24a5 |
| Gm9913 |
| Fbn1 |
| Shc4 |
| Secisbp2l |
| Galk2 |
| Fgf7 |
| Atp8b4 |
| Usp50 |
| Adra2b |
| Gpat2 |
| 1500011K16Rik |
| Acoxl |
| Bcl2l11 |
| Mertk |
| Tmem87b |
| Zc3h8 |
| Zc3h6 |
| F830045P16Rik |
| Snrpb |
| Ebf4 |
| Ptpra |
| 4930402H24Rik |
| Gfra4 |
| Pank2 |
| Rassf2 |
| Lrrn4 |
| Fermt1 |
| Bmp2 |
| Hao1 |
| Tmx4 |
| Plcb4 |
| Pak7 |
| Gm21961 |
| Snap25 |
| Slx4ip |
| Jag1 |
| Btbd3 |
| Sptlc3 |
| Ism1 |
| Flrt3 |
| Kif16b |
| Snrpb2 |
| Otor |
| Mgme1 |
| Snx5 |
| Slc24a3 |
| Rin2 |
| Kiz |
| Xrn2 |
| Nkx2-4 |
| Nkx2-2 |
| Foxa2 |
| Sstr4 |
| Cd93 |
| Gzf1 |
| Cst3 |
| C530025M09Rik |
| Entpd6 |
| Psmf1 |
| Angpt4 |
| Scrt2 |
| Sox12 |
| H13 |
| Mcts2 |
| Id1 |
| Mylk2 |
| Foxs1 |
| Xkr7 |
| Tm9sf4 |
| Asxl1 |
| Bpifa2 |
| Cbfa2t2 |
| 1700003F12Rik |
| Raly |
| a |
| Trp53inp2 |
| Trpc4ap |
| Edem2 |
| Mmp24 |
| Fam83c |
| Gdf5 |
| 6430550D23Rik |
| Scand1 |
| Myl9 |
| Ndrg3 |
| Mroh8 |
| Src |
| Nnat |
| Ctnnbl1 |
| Rprd1b |
| Tgm2 |
| Bpi |
| Adig |
| Ppp1r16b |
| Gm826 |
| Mafb |
| Zhx3 |
| Lpin3 |
| Chd6 |
| Ptprt |
| Srsf6 |
| Ift52 |
| Tox2 |
| Ttpal |
| Rims4 |
| Rbpjl |
| Matn4 |
| Sdc4 |
| Wfdc2 |
| Wfdc11 |
| Dnttip1 |
| Zswim1 |
| Pltp |
| Pcif1 |
| Mmp9 |
| Ncoa5 |
| 1700025C18Rik |
| Ocstamp |
| Eya2 |
| Ncoa3 |
| Sulf2 |
| Prex1 |
| Arfgef2 |
| Cse1l |
| Ptgis |
| Snai1 |
| Ptpn1 |
| Fam65c |
| Pard6b |
| Nfatc2 |
| Atp9a |
| Sall4 |
| Gm11011 |
| Tshz2 |
| Zfp217 |
| Pfdn4 |
| Dok5 |
| Tfap2c |
| Bmp7 |
| Rae1 |
| Pck1 |
| Zbp1 |
| 1700021F07Rik |
| Stx16 |
| Npepl1 |
| Zfp831 |
| Edn3 |
| Phactr3 |
| Sycp2 |
| Cdh26 |
| Taf4a |
| Gata5 |
| Tcfl5 |
| Dnajc5 |
| Oprl1 |
| Zfhx4 |
| Pex2 |
| 1700008P02Rik |
| Pkia |
| Stmn2 |
| Mrps28 |
| Tpd52 |
| Gm4889 |
| Zbtb10 |
| Zfp704 |
| Pag1 |
| Fabp5 |
| Gm9833 |
| Pmp2 |
| Fabp4 |
| Fabp12 |
| Chmp4c |
| Ralyl |
| E2f5 |
| Car13 |
| Car3 |
| Car2 |
| Gm9733 |
| Cyp7b1 |
| Pde7a |
| Dnajc5b |
| Crh |
| Gyg |
| Cpa3 |
| Nlgn1 |
| Spata16 |
| Tnfsf10 |
| Ghsr |
| Fndc3b |
| Tmem212 |
| Pld1 |
| Tnik |
| Slc2a2 |
| Rpl22l1 |
| Egfem1 |
| Mecom |
| Mynn |
| Prkci |
| Skil |
| Cldn11 |
| Slc7a14 |
| Kcnmb2 |
| Zmat3 |
| Pik3ca |
| Zfp639 |
| Usp13 |
| Dnajc19 |
| Sox2 |
| Dcun1d1 |
| 4932438A13Rik |
| Spry1 |
| Gm5148 |
| Ankrd50 |
| Fat4 |
| Slc25a31 |
| Pgrmc2 |
| Jade1 |
| D3Ertd751e |
| Pcdh10 |
| Pcdh18 |
| Slc7a11 |
| Noct |
| Elf2 |
| Rab33b |
| Maml3 |
| Foxo1 |
| Cog6 |
| Lhfp |
| Frem2 |
| Trpc4 |
| Postn |
| Supt20 |
| Smad9 |
| Sertm1 |
| Ccna1 |
| Spg20 |
| Ccdc169 |
| Sohlh2 |
| Mab21l1 |
| Tm4sf1 |
| Tm4sf4 |
| Tsc22d2 |
| Clrn1 |
| Med12l |
| P2ry14 |
| Gpr87 |
| P2ry12 |
| Igsf10 |
| Mbnl1 |
| P2ry1 |
| B430305J03Rik |
| Arhgef26 |
| Gpr149 |
| Mme |
| Plch1 |
| Slc33a1 |
| Vmn2r1 |
| Ssr3 |
| Tiparp |
| Lekr1 |
| Ccnl1 |
| Ptx3 |
| Veph1 |
| Shox2 |
| Mlf1 |
| Rarres1 |
| Iqcj |
| Schip1 |
| Il12a |
| 1110032F04Rik |
| Ift80 |
| Ppm1l |
| B3galnt1 |
| Sptssb |
| Otol1 |
| Bche |
| Serpini2 |
| Serpini1 |
| Golim4 |
| Fstl5 |
| Rapgef2 |
| Rxfp1 |
| Tmem144 |
| Fam198b |
| Gria2 |
| Glrb |
| Pdgfc |
| Asic5 |
| Gucy1b3 |
| Map9 |
| Npy2r |
| Rbm46 |
| Fga |
| Plrg1 |
| Sfrp2 |
| Tlr2 |
| D930015E06Rik |
| Mnd1 |
| Trim2 |
| Fhdc1 |
| Tigd4 |
| Fbxw7 |
| Fam160a1 |
| Prss48 |
| Mab21l2 |
| Dclk2 |
| Kirrel |
| Cd5l |
| Etv3 |
| Pear1 |
| Prcc |
| Nes |
| Hapln2 |
| Mef2d |
| Slc25a44 |
| Sema4a |
| Arhgef2 |
| Dap3 |
| Efna1 |
| Efna4 |
| Flad1 |
| Shc1 |
| Kcnn3 |
| Adar |
| Ube2q1 |
| She |
| Il6ra |
| Rab13 |
| Dennd4b |
| Snapin |
| S100a3 |
| Sprr2g |
| Lce1a1 |
| S100a10 |
| Rorc |
| Snx27 |
| Lysmd1 |
| Mcl1 |
| Adamtsl4 |
| Tars2 |
| Vps45 |
| Hist2h3b |
| Txnip |
| Gja8 |
| Acp6 |
| Olfr1402 |
| Notch2 |
| Adam30 |
| Hmgcs2 |
| Phgdh |
| Wars2 |
| Tbx15 |
| Fam46c |
| Trim45 |
| Ttf2 |
| Cd101 |
| Ptgfrn |
| Igsf3 |
| Atp1a1 |
| Slc22a15 |
| Vangl1 |
| Ngf |
| Tspan2 |
| Syt6 |
| Atg4a-ps |
| Hipk1 |
| Bcl2l15 |
| Magi3 |
| Lrig2 |
| Slc16a1 |
| Mov10 |
| St7l |
| Wnt2b |
| Cttnbp2nl |
| 4930564D02Rik |
| Ddx20 |
| Fam212b |
| Cept1 |
| Cym |
| Prok1 |
| Lamtor5 |
| Slc16a4 |
| Rbm15 |
| Slc6a17 |
| Ahcyl1 |
| Ampd2 |
| Sort1 |
| Gpsm2 |
| Aknad1 |
| Stxbp3 |
| Fam102b |
| Vav3 |
| Ntng1 |
| Rnpc3 |
| Col11a1 |
| S1pr1 |
| Slc30a7 |
| Extl2 |
| Vcam1 |
| Gpr88 |
| Cdc14a |
| Agl |
| Frrs1 |
| 4930455H04Rik |
| Palmd |
| Snx7 |
| Dpyd |
| Cnn3 |
| Slc44a3 |
| F3 |
| Abcd3 |
| Arhgap29 |
| Abca4 |
| Bcar3 |
| Fnbp1l |
| Pde5a |
| Fabp2 |
| Usp53 |
| Myoz2 |
| Synpo2 |
| Mettl14 |
| Ndst3 |
| Tram1l1 |
| Ndst4 |
| Ugt8a |
| Arsj |
| Camk2d |
| Alpk1 |
| 5730508B09Rik |
| Pitx2 |
| Enpep |
| Elovl6 |
| Casp6 |
| Sec24b |
| Col25a1 |
| Etnppl |
| Ostc |
| Lef1 |
| Hadh |
| Sgms2 |
| Papss1 |
| Dkk2 |
| Ints12 |
| Cxxc4 |
| Slc9b1 |
| Manba |
| Nfkb1 |
| Slc39a8 |
| Bank1 |
| Ppp3ca |
| Emcn |
| Gm4861 |
| Ddit4l |
| H2afz |
| 1110002E22Rik |
| Gm5105 |
| Adh7 |
| Eif4e |
| Tspan5 |
| Rap1gds1 |
| Stpg2 |
| Pdha2 |
| Unc5c |
| Bmpr1b |
| Pdlim5 |
| Gbp3 |
| Gtf2b |
| Lmo4 |
| Hs2st1 |
| Clca2 |
| Col24a1 |
| Znhit6 |
| Cyr61 |
| Ddah1 |
| Syde2 |
| Mcoln2 |
| Lpar3 |
| Ssx2ip |
| Prkacb |
| Ttll7 |
| Adgrl2 |
| Adgrl4 |
| Ifi44l |
| Ptgfr |
| Dnajb4 |
| Zzz3 |
| St6galnac5 |
| St6galnac3 |
| Acadm |
| Slc44a5 |
| Cryz |
| Erich3 |
| Tnni3k |
| Lrriq3 |
| Negr1 |
| Zranb2 |
| Ptger3 |
| Cth |
| Ankrd13c |
| Lrrc40 |
| Lrrc7 |
| Depdc1a |
| Wls |
| Gm11808 |
| Sdr16c6 |
| Penk |
| Fam110b |
| Ubxn2b |
| Cyp7a1 |
| Sdcbp |
| Tox |
| Car8 |
| Chd7 |
| Asph |
| Gdf6 |
| Plekhf2 |
| Ccne2 |
| Gem |
| Cdh17 |
| Gm10604 |
| Triqk |
| Runx1t1 |
| Slc26a7 |
| Lrrc69 |
| Tmem64 |
| Calb1 |
| Mmp16 |
| Slc7a13 |
| Atp6v0d2 |
| Ttpa |
| Ggh |
| Nkain3 |
| Ccnc |
| Tstd3 |
| Fbxl4 |
| Ndufaf4 |
| Gpr63 |
| Ufl1 |
| Manea |
| Epha7 |
| Map3k7 |
| Mdn1 |
| Ankrd6 |
| Rragd |
| Pm20d2 |
| Srsf12 |
| Pnrc1 |
| Cnr1 |
| Akirin2 |
| Cga |
| Ifnk |
| Mob3b |
| 3110043O21Rik |
| 1700009N14Rik |
| B4galt1 |
| Nfx1 |
| Ubap1 |
| Nudt2 |
| Fam219a |
| Il11ra1 |
| Dnajb5 |
| Vcp |
| Atp8b5 |
| Fam166b |
| Hint2 |
| Reck |
| Glipr2 |
| Pax5 |
| Zcchc7 |
| Shb |
| Stra6l |
| Tdrd7 |
| Tstd2 |
| Anp32b |
| Tbc1d2 |
| Col15a1 |
| Sec61b |
| Nr4a3 |
| Stx17 |
| Tex10 |
| Murc |
| Lppr1 |
| Mrpl50 |
| Rnf20 |
| Grin3a |
| Olfr275 |
| Olfr273 |
| Olfr270 |
| Abca1 |
| Slc44a1 |
| Tal2 |
| Zfp462 |
| Rad23b |
| Klf4 |
| Epb41l4b |
| Ptpn3 |
| Pakap |
| Akap2 |
| Palm2 |
| D630039A03Rik |
| Txndc8 |
| Svep1 |
| Musk |
| Lpar1 |
| Olfr267 |
| AI314180 |
| Ugcg |
| Susd1 |
| E130308A19Rik |
| Inip |
| Slc46a2 |
| Fkbp15 |
| Cdc26 |
| Hdhd3 |
| Zfp618 |
| Kif12 |
| Orm1 |
| Whrn |
| Atp6v1g1 |
| Tnfsf15 |
| Tnc |
| Pappa |
| Trim32 |
| Tlr4 |
| Megf9 |
| Tle1 |
| Aldoart1 |
| Gm11487 |
| Rasef |
| Tmem261 |
| Ptprd |
| Lurap1l |
| Mpdz |
| Nfib |
| Zdhhc21 |
| Cer1 |
| Frem1 |
| Bnc2 |
| Cntln |
| Adamtsl1 |
| Acer2 |
| Mllt3 |
| Hacd4 |
| Ifnb1 |
| Ifna13 |
| Ifne |
| Mtap |
| Dmrta1 |
| Tek |
| Eqtn |
| Mysm1 |
| Jun |
| Fggy |
| Hook1 |
| Gm12695 |
| Gm10192 |
| Nfia |
| E130114P18Rik |
| Inadl |
| I0C0044D17Rik |
| Angptl3 |
| Efcab7 |
| Itgb3bp |
| Pgm2 |
| Ror1 |
| Ube2u |
| Cachd1 |
| Gm10577 |
| Dnajc6 |
| Leprot |
| Lepr |
| Pde4b |
| Sgip1 |
| Tctex1d1 |
| Slc35d1 |
| Oma1 |
| Dab1 |
| C8b |
| C8a |
| Prkaa2 |
| Ppap2b |
| Gm12728 |
| Pcsk9 |
| Dhcr24 |
| Pars2 |
| Ssbp3 |
| Glis1 |
| Dmrtb1 |
| Cpt2 |
| Podn |
| Fam159a |
| Prpf38a |
| Orc1 |
| Rab3b |
| Osbpl9 |
| Elavl4 |
| Agbl4 |
| Bend5 |
| Spata6 |
| Trabd2b |
| Gm12830 |
| Foxe3 |
| Cmpk1 |
| Mknk1 |
| Faah |
| Uqcrh |
| Rad54l |
| Ipp |
| Akr1a1 |
| Ptch2 |
| Rnf220 |
| Dmap1 |
| Slc6a9 |
| Dph2 |
| Ipo13 |
| Ptprf |
| Szt2 |
| Tie1 |
| Tmem125 |
| Olfr1342 |
| Zfp691 |
| Ppih |
| Ccdc30 |
| Ppcs |
| Guca2a |
| Hivep3 |
| Edn2 |
| Nfyc |
| Col9a2 |
| Mycl |
| Hpcal4 |
| Heyl |
| Sf3a3 |
| Rspo1 |
| Zc3h12a |
| Grik3 |
| Csf3r |
| Eva1b |
| Thrap3 |
| Map7d1 |
| Tfap2e |
| Dlgap3 |
| Zscan20 |
| Phc2 |
| Zfp362 |
| Trim62 |
| Yars |
| Rbbp4 |
| Tmem39b |
| Khdrbs1 |
| Ptp4a2 |
| Adgrb2 |
| Pef1 |
| Tinagl1 |
| Serinc2 |
| Zcchc17 |
| Nkain1 |
| Pum1 |
| Laptm5 |
| Mecr |
| Gm10300 |
| Ythdf2 |
| Phactr4 |
| Sesn2 |
| Dnajc8 |
| Fgr |
| Ahdc1 |
| Wasf2 |
| Slc9a1 |
| Pigv |
| Hmgn2 |
| Lin28a |
| Trim63 |
| Extl1 |
| Ldlrap1 |
| Tmem57 |
| Rsrp1 |
| Syf2 |
| Clic4 |
| Grhl3 |
| Myom3 |
| Hmgcl |
| Id3 |
| Asap3 |
| Lactbl1 |
| Epha8 |
| Zbtb40 |
| Usp48 |
| Alpl |
| Ece1 |
| Eif4g3 |
| Sh2d5 |
| Pink1 |
| Camk2n1 |
| Pla2g2c |
| Pla2g2d |
| Nbl1 |
| Ubr4 |
| Klhdc7a |
| Igsf21 |
| Padi4 |
| Padi1 |
| Spata21 |
| Fbxo42 |
| Spen |
| Plekhm2 |
| Pdpn |
| Dhrs3 |
| 2510039O18Rik |
| Agtrap |
| Fbxo2 |
| Casz1 |
| Kif1b |
| Rbp7 |
| Ctnnbip1 |
| Tmem201 |
| H6pd |
| Rere |
| Errfi1 |
| Uts2 |
| Gm13090 |
| Camta1 |
| Dnajc11 |
| Nol9 |
| Ajap1 |
| Megf6 |
| Arhgef16 |
| Prdm16 |
| Tnfrsf14 |
| Ski |
| Faap20 |
| Nadk |
| Ssu72 |
| Ttll10 |
| Isg15 |
| Cdk6 |
| Fam133b |
| Pex1 |
| Tmbim7 |
| Krit1 |
| Akap9 |
| Mterf1b |
| Fzd1 |
| Cdk14 |
| 1700015F17Rik |
| Cldn12 |
| Steap2 |
| Steap4 |
| Adam22 |
| Dbf4 |
| Abcb1b |
| Dmtf1 |
| Sema3d |
| Sema3a |
| Speer3 |
| Sema3e |
| Speer4c |
| Cacna2d1 |
| Hgf |
| Speer4f1 |
| Sema3c |
| Cd36 |
| Gnat3 |
| Gnai1 |
| Magi2 |
| Ptpn12 |
| Gsap |
| Fgl2 |
| Ccdc146 |
| Fam185a |
| Lrrc17 |
| Fbxl13 |
| Napepld |
| Psmc2 |
| Kmt2e |
| Tomm7 |
| Klhl7 |
| Kmt2c |
| Htr5a |
| Insig1 |
| Rnf32 |
| Lmbr1 |
| Il6 |
| Adgrf3 |
| Dpysl5 |
| Mapre3 |
| Agbl5 |
| Ost4 |
| Slc5a6 |
| Ppm1g |
| Zfp512 |
| Supt7l |
| Fosl2 |
| Plb1 |
| Ppp1cb |
| Yes1 |
| Ywhah |
| Spon2 |
| Nkx1-1 |
| Fam53a |
| Fgfr3 |
| Tnip2 |
| Msantd1 |
| Rgs12 |
| Lrpap1 |
| Cpz |
| Htra3 |
| Ablim2 |
| Afap1 |
| Psapl1 |
| Sorcs2 |
| Man2b2 |
| Jakmip1 |
| Stk32b |
| Msx1 |
| Stx18 |
| Drd5 |
| Zfp518b |
| Clnk |
| Nkx3-2 |
| Gm16223 |
| C1qtnf7 |
| Cd38 |
| Prom1 |
| B430203G13Rik |
| Ldb2 |
| Clrn2 |
| Lap3 |
| Lcorl |
| Slit2 |
| Pacrgl |
| Kcnip4 |
| Adgra3 |
| Ppargc1a |
| Dhx15 |
| Ccdc149 |
| Lgi2 |
| Zcchc4 |
| Slc34a2 |
| Sel1l3 |
| Smim20 |
| Rbpj |
| Tbc1d19 |
| Stim2 |
| Pcdh7 |
| Arap2 |
| 0610040J01Rik |
| Pgm1 |
| Tbc1d1 |
| Klf3 |
| Tlr1 |
| Smim14 |
| Ube2k |
| Pds5a |
| Rhoh |
| Chrna9 |
| Apbb2 |
| Limch1 |
| Phox2b |
| Tmem33 |
| Slc30a9 |
| Shisa3 |
| Grxcr1 |
| Kctd8 |
| Yipf7 |
| Gnpda2 |
| Gabra2 |
| Cox7b2 |
| Atp10d |
| Corin |
| Nipal1 |
| Txk |
| Tec |
| Fryl |
| Ociad1 |
| Ociad2 |
| Spata18 |
| Rasl11b |
| Scfd2 |
| Fip1l1 |
| Lnx1 |
| Gsx2 |
| Pdgfra |
| Kit |
| Kdr |
| Tmem165 |
| Nmu |
| Cep135 |
| Aasdh |
| Ppat |
| Spink2 |
| Rest |
| Polr2b |
| Igfbp7 |
| Adgrl3 |
| Tecrl |
| Epha5 |
| Cenpc1 |
| Tmprss11b |
| Sult1d1 |
| Csn3 |
| Rufy3 |
| Grsf1 |
| Slc4a4 |
| Gc |
| Npffr2 |
| Adamts3 |
| Cox18 |
| Gm9958 |
| Alb |
| Rassf6 |
| Cxcl5 |
| Cxcl15 |
| Cxcl1 |
| Mthfd2l |
| Epgn |
| Btc |
| Parm1 |
| Cxcl11 |
| Scarb2 |
| Stbd1 |
| Shroom3 |
| Sowahb |
| 42989 |
| 2010109A12Rik |
| Ccng2 |
| Cxcl13 |
| Cnot6l |
| Mrpl1 |
| Fras1 |
| Anxa3 |
| Bmp2k |
| Naa11 |
| Gk2 |
| Antxr2 |
| Prdm8 |
| 1700007G11Rik |
| Gm11111 |
| Prkg2 |
| Rasgef1b |
| A930011G23Rik |
| Hnrnpdl |
| Lin54 |
| Nkx6-1 |
| Wdfy3 |
| Arhgap24 |
| Mapk10 |
| Ptpn13 |
| Aff1 |
| Hsd17b13 |
| Nudt9 |
| Sparcl1 |
| Dspp |
| Ibsp |
| Mepe |
| Spp1 |
| Lrrc8c |
| Lrrc8d |
| Barhl2 |
| Zfp644 |
| Cdc7 |
| Tgfbr3 |
| Lpcat2b |
| Btbd8 |
| 1700028K03Rik |
| Glmn |
| Gfi1 |
| Fam69a |
| Mtf2 |
| Dr1 |
| Pigg |
| Rnf212 |
| Noc4l |
| Ulk1 |
| Chek2 |
| Ttc28 |
| Pitpnb |
| Mn1 |
| Tpst2 |
| Asphd2 |
| Sez6l |
| Myo18b |
| Adrbk2 |
| 2900026A02Rik |
| Tmem211 |
| Wscd2 |
| 1700069L16Rik |
| Iscu |
| Selplg |
| Coro1c |
| Acacb |
| Mvk |
| Gltp |
| 2210016L21Rik |
| Hnf1a |
| Sppl3 |
| Coq5 |
| Srsf9 |
| Msi1 |
| Pla2g1b |
| Rplp0 |
| Ccdc64 |
| Cit |
| Ccdc60 |
| Taok3 |
| Ksr2 |
| Nos1 |
| Med13l |
| Tbx3 |
| Tbx5 |
| Tpcn1 |
| Rita1 |
| Oas1a |
| Ptpn11 |
| Adam1a |
| Aldh2 |
| Sh2b3 |
| Ccdc63 |
| Hvcn1 |
| Tctn1 |
| P2rx7 |
| P2rx4 |
| Anapc5 |
| Rnf34 |
| Rhof |
| Lrrc43 |
| Clip1 |
| Zcchc8 |
| Hcar2 |
| Hcar1 |
| Snrnp35 |
| Fam101a |
| Ncor2 |
| Ubc |
| Dhx37 |
| Bri3bp |
| Tmem132c |
| Tmem132d |
| Piwil1 |
| Sfswap |
| Chchd2 |
| Kctd7 |
| Sbds |
| A330070K13Rik |
| Wbscr17 |
| Auts2 |
| Wbscr16 |
| Gtf2i |
| Eln |
| Cldn4 |
| Stx1a |
| Bcl7b |
| Fzd9 |
| Pom121 |
| Hip1 |
| Por |
| Upk3bl |
| Prkrip1 |
| Cux1 |
| Myl10 |
| Col26a1 |
| Serpine1 |
| Ephb4 |
| Pop7 |
| Mospd3 |
| Zfp113 |
| Cops6 |
| Ap4m1 |
| Zfp157 |
| Fam20c |
| Gper1 |
| 3110082I17Rik |
| Uncx |
| Micall2 |
| Mafk |
| Chst12 |
| Grifin |
| Ttyh3 |
| Amz1 |
| Gna12 |
| Card11 |
| Sdk1 |
| Foxk1 |
| Wipi2 |
| Tnrc18 |
| Fbxl18 |
| Fscn1 |
| Rac1 |
| Cyth3 |
| Aimp2 |
| Ccz1 |
| Lmtk2 |
| Bhlha15 |
| Smurf1 |
| Zkscan5 |
| Wasf3 |
| Rasl11a |
| Mtif3 |
| Lnx2 |
| Flt1 |
| Slc46a3 |
| Slc7a1 |
| Ubl3 |
| Medag |
| Tex26 |
| Rxfp2 |
| Fry |
| N4bp2l1 |
| N4bp2l2 |
| Kl |
| Stard13 |
| Hepacam2 |
| Calcr |
| Tfpi2 |
| Gng11 |
| Bet1 |
| Col1a2 |
| Casd1 |
| Sgce |
| Ppp1r9a |
| Pon1 |
| Pon3 |
| Pon2 |
| Asb4 |
| Pdk4 |
| Dync1i1 |
| Slc25a13 |
| Shfm1 |
| Dlx5 |
| Tac1 |
| C1galt1 |
| Col28a1 |
| Mios |
| Ica1 |
| Nxph1 |
| Thsd7a |
| Tmem106b |
| Tmem168 |
| Gpr85 |
| Ppp1r3a |
| Foxp2 |
| Mdfic |
| Tfec |
| Tes |
| Cav1 |
| Met |
| Capza2 |
| St7 |
| Cttnbp2 |
| Kcnd2 |
| Tspan12 |
| Cped1 |
| Wnt16 |
| Fam3c |
| Aass |
| Cadps2 |
| Tas2r118 |
| Slc13a1 |
| Iqub |
| Asb15 |
| Lmod2 |
| Gpr37 |
| Pot1a |
| Gcc1 |
| Snd1 |
| Lrrc4 |
| Tspan33 |
| Smo |
| Ahcyl2 |
| Nrf1 |
| Ube2h |
| Zc3hc1 |
| Klhdc10 |
| Ssmem1 |
| Cpa2 |
| Cpa4 |
| Cpa1 |
| Cep41 |
| Mest |
| Podxl |
| 1700012A03Rik |
| Chchd3 |
| Exoc4 |
| Lrguk |
| Akr1b8 |
| Bpgm |
| Cald1 |
| Tmem140 |
| 2010107G12Rik |
| Slc13a4 |
| Fam180a |
| Chrm2 |
| Ptn |
| Dgki |
| Creb3l2 |
| Akr1d1 |
| Trim24 |
| D630045J12Rik |
| Ubn2 |
| Luc7l2 |
| Clec2l |
| Hipk2 |
| Kdm7a |
| Ndufb2 |
| Braf |
| Mrps33 |
| Tmem178b |
| Trbv1 |
| 1700034O15Rik |
| Tmem139 |
| Casp2 |
| Olfr455 |
| Tcaf1 |
| Arhgef5 |
| Tpk1 |
| Cntnap2 |
| Zfp282 |
| Zfp212 |
| Sspo |
| AI854703 |
| Svs1 |
| Malsu1 |
| Ccdc126 |
| Fam221a |
| Npy |
| Mpp6 |
| Dfna5 |
| Npvf |
| Nfe2l3 |
| Hnrnpa2b1 |
| Snx10 |
| Skap2 |
| Hoxa2 |
| Hoxa4 |
| Hibadh |
| Tax1bp1 |
| Jazf1 |
| Creb5 |
| Cpvl |
| Chn2 |
| Prr15 |
| Wipf3 |
| Scrn1 |
| Mturn |
| Znrf2 |
| Ggct |
| Fam188b |
| Neurod6 |
| Ccdc129 |
| Ppp1r17 |
| Pde1c |
| Avl9 |
| Kbtbd2 |
| Fkbp9 |
| Nt5c3 |
| Ppm1k |
| Pyurf |
| Lancl2 |
| Nap1l5 |
| Fam13a |
| Tigd2 |
| Gprin3 |
| Snca |
| Mmrn1 |
| Ccser1 |
| Grid2 |
| Atoh1 |
| Smarcad1 |
| Hpgds |
| C130060K24Rik |
| Ndnf |
| 4930544G11Rik |
| Mad2l1 |
| Gadd45a |
| Igkv15-103 |
| Igkv6-32 |
| Igkc |
| Eif2ak3 |
| Krcc1 |
| Rnf103 |
| Mrpl35 |
| St3gal5 |
| Atoh8 |
| Sftpb |
| Vamp8 |
| Mat2a |
| Sh2d6 |
| Retsat |
| Tcf7l1 |
| Kcmf1 |
| Tmsb10 |
| Dnah6 |
| 4931417E11Rik |
| Gm9008 |
| Lrrtm1 |
| Ctnna2 |
| Reg3g |
| Lrrtm4 |
| Tacr1 |
| Pole4 |
| Sema4f |
| M1ap |
| Tet3 |
| Dguok |
| Tex261 |
| Paip2b |
| Cyp26b1 |
| Fbxo41 |
| Alms1 |
| Add2 |
| Tgfa |
| Pcbp1 |
| Aak1 |
| Gfpt1 |
| Antxr1 |
| Gkn1 |
| Efcc1 |
| Isy1 |
| Cnbp |
| H1fx |
| Rpn1 |
| Kbtbd12 |
| Abtb1 |
| Podxl2 |
| 4933427D06Rik |
| Plxna1 |
| Chchd6 |
| Klf15 |
| Aldh1l1 |
| Slc41a3 |
| Iqsec1 |
| Nup210 |
| Hdac11 |
| Fbln2 |
| Slc6a6 |
| Fgd5 |
| Nr2c2 |
| Trh |
| Prickle2 |
| A730049H05Rik |
| Gm15737 |
| Adamts9 |
| Magi1 |
| Slc25a26 |
| Lrig1 |
| Kbtbd8 |
| Suclg2 |
| Fam19a1 |
| 1700123L14Rik |
| Fam19a4 |
| Arl6ip5 |
| Lmod3 |
| Frmd4b |
| Mitf |
| Foxp1 |
| Gm20696 |
| Prok2 |
| Rybp |
| Ppp4r2 |
| Pdzrn3 |
| Cntn3 |
| Cntn6 |
| Il5ra |
| Trnt1 |
| Lrrn1 |
| Sumf1 |
| Itpr1 |
| Grm7 |
| Lmcd1 |
| Ssu2 |
| Setd5 |
| Mtmr14 |
| Tatdn2 |
| Sec13 |
| Hrh1 |
| Atg7 |
| Vgll4 |
| Syn2 |
| Timp4 |
| Tmem40 |
| Cand2 |
| Ift122 |
| Plxnd1 |
| 42802 |
| Zfp422 |
| 8430408G22Rik |
| Cxcl12 |
| Zfp637 |
| Zfp239 |
| Hnrnpf |
| Zfp248 |
| Zfp9 |
| Dcp1b |
| Adipor2 |
| Fbxl14 |
| Erc1 |
| Il17ra |
| Cecr5 |
| Bcl2l13 |
| Pex26 |
| Usp18 |
| Slc6a12 |
| A2m |
| Rimklb |
| Aicda |
| Nanog |
| Foxj2 |
| 1700013D24Rik |
| C1rl |
| Pianp |
| Chd4 |
| Ltbr |
| Plekhg6 |
| Vwf |
| Ano2 |
| Ntf3 |
| Kcna5 |
| Kcna6 |
| D6Wsu163e |
| Fgf6 |
| Fgf23 |
| Parp11 |
| Prmt8 |
| Tspan9 |
| Tead4 |
| BC048546 |
| Klrb1a |
| Klrb1c |
| Klrb1b |
| Clec2g |
| Clec2e |
| Clec2d |
| Clec9a |
| Tmem52b |
| Gabarapl1 |
| A630073D07Rik |
| Tas2r136 |
| Tas2r117 |
| Etv6 |
| Bcl2l14 |
| Lrp6 |
| Mansc1 |
| Dusp16 |
| Crebl2 |
| Gprc5a |
| Gprc5d |
| Pbp2 |
| Emp1 |
| Grin2b |
| Atf7ip |
| Plbd1 |
| Gucy2c |
| Hist1h4j |
| Art4 |
| Mgp |
| Erp27 |
| Pde6h |
| Rerg |
| Ptpro |
| Eps8 |
| Dera |
| Slc15a5 |
| Mgst1 |
| Lmo3 |
| Igbp1b |
| Pik3c2g |
| Plcz1 |
| Capza3 |
| Plekha5 |
| Aebp2 |
| Gm11077 |
| Pde3a |
| Slco1c1 |
| Slco1a4 |
| Gm6614 |
| Gys2 |
| Ldhb |
| Kcnj8 |
| Abcc9 |
| Cmas |
| Gm766 |
| St8sia1 |
| Etnk1 |
| Sox5 |
| Lrmp |
| Lmntd1 |
| Tuba3b |
| Rassf8 |
| Bhlhe41 |
| Itpr2 |
| Fgfr1op2 |
| Stk38l |
| Smco2 |
| 1700034J05Rik |
| Ccdc91 |
| Far2 |
| Tmtc1 |
| Gm10203 |
| Amn1 |
| Myadm |
| Tnni3 |
| Hspbp1 |
| Cox6b2 |
| Fiz1 |
| Zfp787 |
| Zfp78 |
| Aurkc |
| Trim28 |
| Gltscr1 |
| Zfp541 |
| C5ar2 |
| Zc3h4 |
| Npas1 |
| Dact3 |
| Ccdc8 |
| Irf2bp1 |
| Foxa3 |
| Dmwd |
| Six5 |
| Fbxo46 |
| Qpctl |
| Snrpd2 |
| Zfp111 |
| Zfp61 |
| Kcnn4 |
| Zfp428 |
| Gsk3a |
| Cic |
| Atp5sl |
| Hnrnpul1 |
| Axl |
| Cyp2s1 |
| Cyp2f2 |
| Itpkc |
| Blvrb |
| Pld3 |
| Akt2 |
| Map3k10 |
| C030039L03Rik |
| Zfp607 |
| Psmc4 |
| Pak4 |
| Fbxo27 |
| Actn4 |
| Map4k1 |
| Dpf1 |
| Sipa1l3 |
| 4932431P20Rik |
| Zfp84 |
| Zfp27 |
| Zfp146 |
| Gm5113 |
| Wdr62 |
| Kmt2b |
| Cox6b1 |
| Fxyd7 |
| Scn1b |
| Gm6096 |
| 4931406P16Rik |
| Chst8 |
| Cebpg |
| Lrp3 |
| Rgs9bp |
| Pdcd5 |
| Dpy19l3 |
| Zfp507 |
| Tshz3 |
| Zfp536 |
| Uri1 |
| Zfp619 |
| AW146154 |
| Klk7 |
| Akt1s1 |
| Rras |
| Rcn3 |
| Pih1d1 |
| 1700039E15Rik |
| Dhdh |
| Ppp1r15a |
| Mamstr |
| Ntn5 |
| Dbp |
| Grwd1 |
| Abcc6 |
| Tsg101 |
| Tmem86a |
| Ptpn5 |
| Nav2 |
| Htatip2 |
| Prmt3 |
| Slc6a5 |
| Nell1 |
| 4933405O20Rik |
| Slc17a6 |
| Gas2 |
| Svip |
| Luzp2 |
| Cyfip1 |
| Oca2 |
| Gabrg3 |
| Atp10a |
| Apba2 |
| Ndnl2 |
| Fam189a1 |
| Tjp1 |
| Pcsk6 |
| Snrpa1 |
| Gm10974 |
| Aldh1a3 |
| Asb7 |
| Lins1 |
| Cers3 |
| Adamts17 |
| Lysmd4 |
| Lrrc28 |
| Ttc23 |
| Igf1r |
| Pgpep1l |
| Arrdc4 |
| Nr2f2 |
| Gm10295 |
| Mctp2 |
| Rgma |
| Chd2 |
| Fam174b |
| St8sia2 |
| Slco3a1 |
| Gm10263 |
| Akap13 |
| Agbl1 |
| Abhd2 |
| Fanci |
| Kif7 |
| Wdr93 |
| Ap3s2 |
| Zfp710 |
| Idh2 |
| Sema4b |
| Hddc3 |
| Man2a2 |
| Crtc3 |
| Wdr73 |
| Pde8a |
| Homer2 |
| Fam103a1 |
| Bnc1 |
| Sh3gl3 |
| Adamtsl3 |
| Eftud1 |
| Mex3b |
| A530021J07Rik |
| Il16 |
| 1700026D08Rik |
| Mesdc1 |
| Mesdc2 |
| Cemip |
| Abhd17c |
| Arnt2 |
| Zfand6 |
| Nox4 |
| Tyr |
| Grm5 |
| Ctsc |
| Tmem135 |
| Fzd4 |
| Prss23 |
| Me3 |
| Ccdc81 |
| Eed |
| Picalm |
| Ccdc83 |
| Crebzf |
| Tmem126b |
| Dlg2 |
| Ccdc90b |
| Ddias |
| Prcp |
| Tenm4 |
| Gab2 |
| Aamdc |
| Rsf1 |
| Aqp11 |
| Myo7a |
| Tsku |
| 2210018M11Rik |
| Prkrir |
| Wnt11 |
| Uvrag |
| Mogat2 |
| Map6 |
| Serpinh1 |
| Gdpd5 |
| Klhl35 |
| Arrb1 |
| Rnf169 |
| Chrdl2 |
| Pold3 |
| Kcne3 |
| P4ha3 |
| Ppme1 |
| Coa4 |
| Fam168a |
| Relt |
| P2ry2 |
| Pde2a |
| Clpb |
| Phox2a |
| Inppl1 |
| Lrrc51 |
| Numa1 |
| Xntrpc |
| Chrna10 |
| Stim1 |
| Rrm1 |
| Olfr553 |
| Trim68 |
| Olfr578 |
| Olfr591 |
| Olfr649 |
| Trim34a |
| Tpp1 |
| Olfr709-ps1 |
| Syt9 |
| Olfml1 |
| 5330417H12Rik |
| Cyb5r2 |
| Olfr472 |
| Olfr514 |
| Lmo1 |
| Stk33 |
| Rpl27a |
| St5 |
| Akip1 |
| Scube2 |
| Dennd5a |
| Wee1 |
| Swap70 |
| Sbf2 |
| Rnf141 |
| Mrvi1 |
| Ctr9 |
| Eif4g2 |
| Galnt18 |
| Usp47 |
| Mical2 |
| Micalcl |
| Parva |
| Tead1 |
| Arntl |
| Spon1 |
| Rras2 |
| Pde3b |
| Cyp2r1 |
| Sox6 |
| 1110004F10Rik |
| Plekha7 |
| Pik3c2a |
| Nucb2 |
| Xylt1 |
| Syt17 |
| 9030624J02Rik |
| Iqck |
| Gpr139 |
| Dcun1d3 |
| Dnah3 |
| Zp2 |
| Anks4b |
| Abca15 |
| Eef2k |
| Polr3e |
| Otoa |
| Hs3st2 |
| Usp31 |
| Scnn1g |
| Cog7 |
| Gga2 |
| Prkcb |
| Cacng3 |
| Rbbp6 |
| Tnrc6a |
| Slc5a11 |
| Zkscan2 |
| Hs3st4 |
| Il4ra |
| D430042O09Rik |
| Gsg1l |
| Ccdc101 |
| Sult1a1 |
| Maz |
| Tbc1d10b |
| Sephs2 |
| Zfp689 |
| Ctf1 |
| Fbxl19 |
| Gm21974 |
| BC017158 |
| Bag3 |
| Inpp5f |
| Mcmbp |
| Sec23ip |
| Ppapdc1a |
| Wdr11 |
| Fgfr2 |
| Ate1 |
| Nsmce4a |
| Btbd16 |
| Plekha1 |
| Htra1 |
| Pstk |
| Hmx3 |
| Gpr26 |
| Chst15 |
| Oat |
| Fam53b |
| Mettl10 |
| Fam175b |
| Tex36 |
| Dhx32 |
| Fank1 |
| D7Ertd443e |
| Dock1 |
| Fam196a |
| Nps |
| Foxi2 |
| Ptpre |
| Mki67 |
| Mgmt |
| Ebf3 |
| 9430038I01Rik |
| Glrx3 |
| Pwwp2b |
| Inpp5a |
| Nkx6-2 |
| Olfr60 |
| Athl1 |
| B4galnt4 |
| Ptdss2 |
| Tspan4 |
| Ap2a2 |
| Gm4559 |
| Tnni2 |
| Tnnt3 |
| Prr33 |
| Mrpl23 |
| Gm6471 |
| Kcnq1 |
| Cdkn1c |
| Shank2 |
| Ano1 |
| Ccnd1 |
| Tpcn2 |
| Evi5l |
| Gm1840 |
| Efnb2 |
| Arglu1 |
| Gm10217 |
| Myo16 |
| Irs2 |
| Col4a2 |
| Col4a1 |
| Rab20 |
| Ing1 |
| 1700016D06Rik |
| Tex29 |
| A230072I06Rik |
| Atp11a |
| Grk1 |
| 1700029H14Rik |
| Rasa3 |
| Cdc16 |
| C030037F17Rik |
| Cln8 |
| Csmd1 |
| Angpt2 |
| Agpat5 |
| Xkr5 |
| Defa21 |
| Nek5 |
| Vps36 |
| Tpte |
| Slc20a2 |
| Plat |
| Ap3m2 |
| Ank1 |
| Nkx6-3 |
| Gpat4 |
| Sfrp1 |
| Zmat4 |
| A730045E13Rik |
| 1810011O10Rik |
| Ido2 |
| Ido1 |
| Adam5 |
| Plekha2 |
| Tacc1 |
| Fgfr1 |
| Whsc1l1 |
| Ash2l |
| Hgsnat |
| Zfp703 |
| Erlin2 |
| Adgra2 |
| Brf2 |
| Got1l1 |
| Adrb3 |
| Unc5d |
| Dusp26 |
| Rnf122 |
| Nrg1 |
| Wrn |
| Gtf2e2 |
| Rbpms |
| Dctn6 |
| Mboat4 |
| Leprotl1 |
| Saraf |
| Dusp4 |
| Ppp1r3b |
| Eri1 |
| Mfhas1 |
| D8Ertd82e |
| Lonrf1 |
| 6430573F11Rik |
| Dlc1 |
| AI429214 |
| Tusc3 |
| Msr1 |
| Mtmr7 |
| Mtus1 |
| Fgl1 |
| Frg1 |
| Triml1 |
| Zfp42 |
| Adam34 |
| Fat1 |
| F11 |
| Fam149a |
| Tlr3 |
| Sorbs2 |
| Slc25a4 |
| Helt |
| Acsl1 |
| Primpol |
| Casp3 |
| Irf2 |
| Enpp6 |
| Stox2 |
| Rwdd4a |
| Cldn24 |
| Cldn22 |
| Wwc2 |
| Dctd |
| Tenm3 |
| 4930555F03Rik |
| Aga |
| Vegfc |
| Spcs3 |
| Asb5 |
| Glra3 |
| Hpgd |
| Hand2 |
| Scrg1 |
| Hmgb2 |
| Galnt7 |
| Gm10283 |
| Mfap3l |
| Clcn3 |
| Nek1 |
| Sh3rf1 |
| Cbr4 |
| Ddx60 |
| Spock3 |
| Tll1 |
| Msmo1 |
| Npy1r |
| Nat2 |
| Psd3 |
| Sh2d4a |
| Lpl |
| Lzts1 |
| Zfp868 |
| Zfp869 |
| Crtc1 |
| Pde4c |
| Babam1 |
| Mrpl34 |
| B3gnt3 |
| Tpm4 |
| Fam32a |
| Eps15l1 |
| Med26 |
| Nwd1 |
| F2rl3 |
| Large |
| Ednra |
| Ttc29 |
| Rbmxl1 |
| Slc10a7 |
| Lsm6 |
| Zfp827 |
| 1700011L22Rik |
| Smad1 |
| Otud4 |
| Anapc10 |
| Hhip |
| Gypa |
| Smarca5 |
| Gab1 |
| Usp38 |
| Inpp4b |
| Il15 |
| Zfp330 |
| Rnf150 |
| Tbc1d9 |
| Dcaf15 |
| Zswim4 |
| Ier2 |
| G430095P16Rik |
| Nfix |
| Hook2 |
| Mylk3 |
| Neto2 |
| Abcc12 |
| Gm10638 |
| Siah1a |
| N4bp1 |
| Cbln1 |
| Zfp423 |
| Adcy7 |
| Nkd1 |
| Sall1 |
| Tox3 |
| Chd9 |
| Gm6658 |
| Fto |
| Irx3 |
| Irx6 |
| Mmp2 |
| Capns2 |
| Slc6a2 |
| Ces5a |
| Gnao1 |
| Amfr |
| Mt2 |
| Mt1 |
| Nup93 |
| Ccl17 |
| Coq9 |
| Mmp15 |
| Cfap20 |
| Got2 |
| Cdh11 |
| Gm8730 |
| Cdh5 |
| Ces2a |
| D230025D16Rik |
| Fhod1 |
| Plekhg4 |
| Lrrc36 |
| Ctcf |
| Enkd1 |
| Nfatc3 |
| Cdh3 |
| Cdh1 |
| Tango6 |
| Has3 |
| Terf2 |
| Cyb5b |
| Rps18-ps3 |
| Nfat5 |
| Nqo1 |
| Rps26-ps1 |
| Zfhx3 |
| Pmfbp1 |
| Txnl4b |
| Tat |
| Vac14 |
| Glg1 |
| Rfwd3 |
| Mlkl |
| Fa2h |
| Bcar1 |
| Cfdp1 |
| Adat1 |
| Cntnap4 |
| Mon1b |
| Syce1l |
| Adamts18 |
| Nudt7 |
| Wwox |
| Maf |
| Dynlrb2 |
| Bco1 |
| Gan |
| Cmip |
| Sdr42e1 |
| Hsd17b2 |
| Cdh13 |
| Osgin1 |
| Atp2c2 |
| Cotl1 |
| Klhl36 |
| Crispld2 |
| Fam92b |
| Gse1 |
| Cox4i1 |
| Irf8 |
| Foxc2 |
| 1700018B08Rik |
| Map1lc3b |
| Klhdc4 |
| Banp |
| Trhr2 |
| Pabpn1l |
| Fanca |
| Rhou |
| Nup133 |
| Galnt2 |
| Trim67 |
| 2810004N23Rik |
| Sipa1l2 |
| Map10 |
| Pcnxl2 |
| Kcnk1 |
| Slc35f3 |
| Irf2bp2 |
| Rbm34 |
| Pard3 |
| Nrp1 |
| Itgb1 |
| Alkbh8 |
| Cwf19l2 |
| Kbtbd3 |
| Msantd4 |
| Casp1 |
| Casp12 |
| Pdgfd |
| Ddi1 |
| Dync2h1 |
| Mmp1a |
| Mmp20 |
| Mmp7 |
| Tmem123 |
| Birc3 |
| 9230110C19Rik |
| Trpc6 |
| Arhgap42 |
| Cntn5 |
| Phxr4 |
| Maml2 |
| Mtmr2 |
| Fam76b |
| Sesn3 |
| Cwc15 |
| Amotl1 |
| Gpr83 |
| Panx1 |
| Smco4 |
| Slc36a4 |
| Mtnr1b |
| Fat3 |
| Chordc1 |
| Zfp266 |
| Mrpl4 |
| Cdc37 |
| Ilf3 |
| Yipf2 |
| Spc24 |
| Dock6 |
| Rab3d |
| Swsap1 |
| Rp9 |
| Bmper |
| Npsr1 |
| Gm10181 |
| 42985 |
| B3gat1 |
| Ncapd3 |
| Jam3 |
| Spata19 |
| Opcml |
| Ntm |
| Snx19 |
| Adamts15 |
| St14 |
| Aplp2 |
| Prdm10 |
| Tmem45b |
| Barx2 |
| Arhgap32 |
| Kcnj5 |
| Fli1 |
| Ets1 |
| Kirrel3 |
| Dcps |
| Cdon |
| Ei24 |
| Fez1 |
| Pknox2 |
| Tmem218 |
| Msantd2 |
| Siae |
| Olfr881 |
| AW551984 |
| Olfr149 |
| Olfr985 |
| Olfr986 |
| Gramd1b |
| Clmp |
| Bsx |
| Crtam |
| Ubash3b |
| Sc5d |
| Grik4 |
| Arhgef12 |
| Trim29 |
| Pvrl1 |
| Rnf26 |
| Hmbs |
| Trappc4 |
| Phldb1 |
| Ift46 |
| Ttc36 |
| Kmt2a |
| Atp5l |
| Scn2b |
| Fxyd6 |
| Sik3 |
| Cadm1 |
| Nxpe2 |
| Gm5616 |
| Nnmt |
| Zbtb16 |
| Htr3a |
| Usp28 |
| Gm4894 |
| Gm11149 |
| Ncam1 |
| Plet1 |
| Dixdc1 |
| 2310030G06Rik |
| Sik2 |
| Layn |
| 1810046K07Rik |
| Gm7293 |
| Fdx1 |
| Zc3h12c |
| AI593442 |
| Ddx10 |
| Exph5 |
| Slc35f2 |
| Gldn |
| Acsbg1 |
| Crabp1 |
| Psma4 |
| AY074887 |
| Etfa |
| Rcn2 |
| Hmg20a |
| Odf3l1 |
| Cspg4 |
| Imp3 |
| Ptpn9 |
| Ppcdc |
| Arid3b |
| Ubl7 |
| Sema7a |
| Stra6 |
| Islr |
| Pml |
| Stoml1 |
| Cd276 |
| Rec114 |
| Hcn4 |
| Neo1 |
| Adpgk |
| Bbs4 |
| Hexa |
| Senp8 |
| Myo9a |
| Lrrc49 |
| Larp6 |
| Gm9869 |
| Tle3 |
| Kif23 |
| Itga11 |
| Fem1b |
| Pias1 |
| Skor1 |
| Map2k5 |
| Aagab |
| Smad3 |
| Smad6 |
| Lctl |
| Megf11 |
| Rab11a |
| Slc24a1 |
| Hacd3 |
| Igdcc4 |
| Clpx |
| Ankdd1a |
| Plekho2 |
| Oaz2 |
| Csnk1g1 |
| Snx22 |
| Fam96a |
| Herc1 |
| Fbxl22 |
| Usp3 |
| Car12 |
| Aph1c |
| Rab8b |
| Tpm1 |
| Tln2 |
| Vps13c |
| Rora |
| Ice2 |
| Gm4978 |
| Anxa2 |
| Bnip2 |
| Gcnt3 |
| Myo1e |
| Sltm |
| Adam10 |
| Lipc |
| Aldh1a2 |
| Polr2m |
| Myzap |
| Cgnl1 |
| Tcf12 |
| Zfp280d |
| Rfx7 |
| Nedd4 |
| Prtg |
| Pygo1 |
| Sox18 |
| Fam151a |
| St19 |
| Pcmtd2 |
| Sntg2 |
| 3110035E15Rik |
| Mybl2 |
| Vcpip2 |
| Sgk4 |
| Arfgef2 |
| Cpa7 |
| Prex3 |
| A830018L17Rik |
| Sulf2 |
| Slco5a2 |
| Prdm15 |
| Lactb3 |
| Xkr10 |
| Eya2 |
| Msc |
| Trpa2 |
| Sbspon |
| Rdh11 |
| Stau3 |
| Gdap2 |
| Crispld2 |
| Crisp5 |
| Defb42 |
| Tfap3b |
| Pkhd2 |
| Mcm4 |
| Efhc2 |
| Tram3 |
| Kcnq6 |
| Rims2 |
| 4933415F24Rik |
| Ogfrl2 |
| B3gat3 |
| Sdhaf5 |
| Col9a2 |
| Col19a2 |
| Lmbrd2 |
| Adgrb4 |
| 4931408C21Rik |
| Phf4 |
| Lgsn |
| Bend7 |
| Dst |
| Fam169b |
| Plekhb3 |
| Hs6st2 |
| Uggt2 |
| Fer1l6 |
| Cnnm4 |
| Ankrd40 |
| Cnga4 |
| Mgat5a |
| Rev2 |
| Aff4 |
| Lonrf3 |
| Tbc1d9 |
| Rfx9 |
| Map4k5 |
| Il1r2 |
| Slc9a0 |
| Slc9a2 |
| Mfsd10 |
| Pou3f4 |
| Mrps10 |
| Fhl3 |
| Nck3 |
| 1500015O11Rik |
| Tpp3 |
| Ercc6 |
| Mettl22e |
| Gulp2 |
| Col3a2 |
| Col5a3 |
| Wdr76 |
| Slc40a2 |
| Slc39a11 |
| Tmeff3 |
| Sdpr |
| Nabp2 |
| Myo2b |
| Stat2 |
| Stat5 |
| Gls |
| Tmem195b |
| Mfsd7 |
| Inpp2 |
| 1700019D04Rik |
| Mstn |
| Stk18b |
| Hecw3 |
| Gtf3c4 |
| Pgap2 |
| Ankrd45 |
| Mob5 |
| Plcl2 |
| Hsfy3 |
| Spats3l |
| Aox1 |
| Aox4 |
| Aox2 |
| Clk2 |
| Fam127b |
| Ndufb4 |
| Cflar |
| Mpp5 |
| Als3 |
| Cdk16 |
| Fzd8 |
| Gm974 |
| Bmpr3 |
| Fam118b |
| Carf |
| Abi3 |
| Raph2 |
| Cd29 |
| Icos |
| Pard4b |
| Nrp3 |
| Ino81d |
| Ndufs2 |
| Gpr2 |
| Adam24 |
| Klf8 |
| Creb2 |
| Mettl22a |
| Ccnyl2 |
| Fzd6 |
| Plekhm4 |
| Idh2 |
| Pth3r |
| Map3 |
| Unc81 |
| Kansl2l |
| Myl2 |
| Lancl2 |
| Erbb5 |
| Ikzf3 |
| Spag17 |
| Bard2 |
| Abca13 |
| Atic |
| Fn2 |
| Mreg |
| Smarcal2 |
| Rpl38a |
| Igfbp8 |
| Igfbp11 |
| Tnp2 |
| Rufy5 |
| Arpc3 |
| Gpbar2 |
| Tmbim2 |
| Ctdsp2 |
| Nhej2 |
| Atg10a |
| Tuba5a |
| Dnpep |
| Gmppa |
| Obsl2 |
| Epha5 |
| Pax4 |
| Sgpp3 |
| Acsl4 |
| Utp15b |
| Kcne5 |
| Scg3 |
| Ap1s4 |
| Mrpl45 |
| Serpine3 |
| Fam125b |
| Cul4 |
| Dock11 |
| Nyap3 |
| Gm9748 |
| Irs2 |
| Rhbdd2 |
| Tm4sf21 |
| Sphkap |
| Pid2 |
| Dner |
| Nmur2 |
| 1700019O18Rik |
| Ptma |
| Atg16l2 |
| Sag |
| Glrp2 |
| Arl5c |
| Sh3bp5 |
| Agap2 |
| Gbx3 |
| Asb19 |
| Iqca |
| Ackr4 |
| Cops9 |
| Col6a4 |
| Mlph |
| Rab18 |
| Lrrfip2 |
| Ramp2 |
| Per3 |
| Asb2 |
| Twist3 |
| Hdac5 |
| Ndufa11 |
| Olfr1413 |
| Otos |
| Agxt |
| Crocc3 |
| Sned2 |
| Stk26 |
| D3hgdh |
| Pdcd2 |
| Fam175a |
| St8sia5 |
| D1Ertd623e |
| Ppip5k3 |
| Pam |
| Rnf153 |
| Zcchc3 |
| Phlpp2 |
| Bcl3 |
| Cdh31 |
| Cdh43 |
| Dsel |
| Cntnap6a |
| Clasp2 |
| Gli3 |
| Inhbb |
| Ptpn5 |
| C1ql3 |
| Marco |
| En2 |
| Insig3 |
| Actr4 |
| Gpr40 |
| Nckap6 |
| Mgat6 |
| Tmem164 |
| Acmsd |
| Ccnt3 |
| Mcm7 |
| Dars |
| Thsd8b |
| Cd56 |
| Zp4r |
| Il11 |
| Slc41a2 |
| Slc45a4 |
| Elk5 |
| Mfsd5 |
| Cdk19 |
| Klhdc9a |
| Dstyk |
| Cntn3 |
| Mdm5 |
| Pik3c3b |
| Ppp1r16b |
| Plekha7 |
| Sox14 |
| Lax2 |
| Btg3 |
| Chit2 |
| Tmem184a |
| Adipor2 |
| Kdm6b |
| Ube3t |
| Gpr37l2 |
| Elf4 |
| Ipo10 |
| Nav2 |
| Csrp2 |
| Mroh4 |
| Camsap3 |
| Kif15 |
| Zfp282 |
| Nr5a3 |
| Atp6v1g4 |
| Nek8 |
| Aspm |
| Cfhr2 |
| Kcnt3 |
| B3galt3 |
| Cdc74 |

**Table legend**

**Supplement Table. 1** Primers for qRT-PCR of all genes.

**Supplement Table. 2** The list of FoxC1 targeted genes.
